# Synergistic amelioration of cholestatic liver fibrosis by combined total astragalus saponins and AAV8.*Numb*-Exon3 through modulation of hepatic progenitor cell differentiation

**DOI:** 10.3389/fphar.2026.1762397

**Published:** 2026-04-23

**Authors:** Mengyao Zong, Yannan Xu, Feifei Xing, Danyang Wang, Xinrui Zheng, Shihao Zhang, Junyi Zhan, Jiamei Chen, Gaofeng Chen, Wei Liu, Ping Liu, Yongping Mu

**Affiliations:** Shuguang Hospital Affiliated to Shanghai University of Traditional Chinese Medicine (TCM), Institute of Liver Diseases, Shanghai University of TCM, Key Laboratory of Liver and Kidney Disease of the Ministry of Education, Clinical Key Laboratory of TCM of Shanghai, Shanghai, China

**Keywords:** cholestatic liver fibrosis, ductular reaction, hepatic progenitor, Numb-Exon3, total astragalus saponins

## Abstract

**Background:**

Despite our previous findings that both total astragalus saponins (TAS) and hepatic AAV8.*Numb*-Exon3 overexpression (AAV8.*Numb*-Ex3^OE^) are effective against cholestatic liver fibrosis (CLF), the synergistic effect and mechanism of their combination remain unknown. This study was designed to investigate this combination therapy and elucidate its underlying molecular mechanisms.

**Methods:**

A rat model of CLF was induced by bile duct ligation and subsequently treated with combination therapy. Evaluations included serum biochemical parameters, liver histopathology, hepatic hydroxyproline (Hyp) content, expression levels of key molecules in the Notch signaling pathway, hepatic progenitor cells (HPCs) differentiation, ductular reaction (DR), and liver regeneration. *In vitro*, WB-F344 cells were induced toward a cholangiocyte-like phenotype, and the influence of TAS on HPCs differentiation was assessed following LV.*Numb*-Ex3 transfection.

**Results:**

The combination therapy more effectively improved serum biochemistry, reduced hepatic inflammation and collagen deposition, and lowered hepatic Hyp content compared to TAS or AAV8.*Numb*-Ex3^OE^ monotherapy. Mechanistically, the combination therapy further suppressed differentiation of HPCs into cholangiocytes and inhibited DR, while promoting HPC-to-hepatocyte differentiation and enhancing liver regeneration.

**Conclusion:**

The combination of TAS and AAV8.*Numb*-Ex3^OE^ exerts a synergistic anti-fibrotic effect in CLF by redirecting HPCs differentiation fate. These findings provide experimental support for the development of novel combinatorial strategies against liver fibrosis.

## 1 Introduction

Cholestatic liver fibrosis (CLF) is a progressive hepatic disorder characterized by biliary injury as the core pathological feature, which can advance to cirrhosis, liver failure, and even hepatocellular carcinoma. The etiology of CLF involves genetic predispositions (e.g., Alagille syndrome), autoimmune dysregulation (e.g., primary biliary cholangitis [PBC] and primary sclerosing cholangitis [PSC]), and biliary tract obstruction (e.g., secondary to chronic pancreatitis), all of which disrupt bile synthesis, secretion, or excretion (Mayo, 2022). Chronic cholestasis initiates key pathological events, including ductular reaction (DR), activation of hepatic stellate cells (HSCs), and impaired hepatocyte regeneration (Fabris et al., 2019; Koyama and Brenner, 2017). Current first-line therapies for PBC, such as ursodeoxycholic acid (UDCA) and obeticholic acid, often fail to halt disease progression (Corpechot et al., 2000; Nevens et al., 2016). Our previous work identified total astragalus saponins (TAS), a bioactive extract from *Astragalus membranaceus* (Fisch.) Bge. *var. mongholicus* (Bge.) Hsiao, as a promising anti-fibrotic agent. TAS ameliorates CLF by suppressing the Notch signaling-mediated the differentiation of hepatic progenitor cells (HPCs) into cholangiocytes, a key driver of DR, and by upregulating hepatic expression of Numb, a negative regulator of the Notch pathway (Yongping et al., 2015).

Numb is a key cell-fate determinant that influences differentiation through asymmetric segregation during cell division (Loeffler et al., 2019). Previous studies from our group revealed a reduction of approximately 73% in hepatic Numb protein levels in PBC-cirrhotic tissues relative to healthy controls. This observation was consistently reproduced in bile duct ligation (BDL)-induced models of CLF (Xu et al., 2023; Zhang et al., 2016). Numb-knockout CLF models exhibited aggravated periportal HPCs proliferation, elevated expression of the DR marker CK19, and increased hepatic collagen deposition (Shu et al., 2021), underscoring Numb deficiency as a pivotal contributor to CLF pathogenesis.

The functional diversity of Numb is further modulated by alternative splicing. In mammals, splicing of Exon 3 (Ex3) and 9 generates four majors isoforms (Numb1-4), with functional differences largely attributed to the Ex3-encoded region. Ex3 codes for an 11-amino-acid segment in the N-terminal phosphotyrosine-binding (PTB) domain, giving rise to long (Numb-PTB_L_, isoforms 1/2) and short (Numb-PTB_S_, isoforms 3/4) variants. The PTB domain mediates protein interactions, membrane localization, and endocytosis (Confalonieri et al., 2019). Crucially, only isoforms with an intact PTB domain (i.e., Numb-PTB_L_) can bind E3 ubiquitin ligases such as LNX or ITCH to promote Notch ubiquitination (Nie et al., 2004; McGill and McGlade, 2003). Moreover, the Ex3-encoded segment is essential for Numb to interact with Mdm2, thereby blocking Mdm2-mediated p53 degradation and maintaining p53 stability (Colaluca et al., 2018; Colaluca et al., 2008). Loss of Numb-PTB_L_ isoforms disrupts p53 activity, leading to aberrant stem cell self-renewal and tumorigenesis. Accordingly, Numb-PTB_L_ isoforms exert tumor-suppressive effects in hepatocellular carcinoma, whereas Numb-PTB_S_ variants may promote oncogenesis (Kim and Ronai, 2018; Machida, 2020; Su et al., 2023). These observations establish the PTB domain, and specifically the Ex3-encoded region, as a critical determinant of Numb’s role in governing cell fate and tissue repair.

Our preclinical data indicate that AAV8-mediated *Numb*-Ex3 delivery alleviates CLF progression (the data has been published in another journal). Building on these findings, the present study investigation the combined effect of TAS and AAV8.*Numb*-Ex3. We demonstrate that this combination therapy superior anti-fibrotic efficacy compared to either monotherapy, achieved through synergistic inhibition of DR and HSC activation, coupled with promoted the differentiation of HPCs into hepatocytes.

## 2 Materials and methods

### 2.1 Preparation and chemical analysis of TAS

**FIGURE 1:**
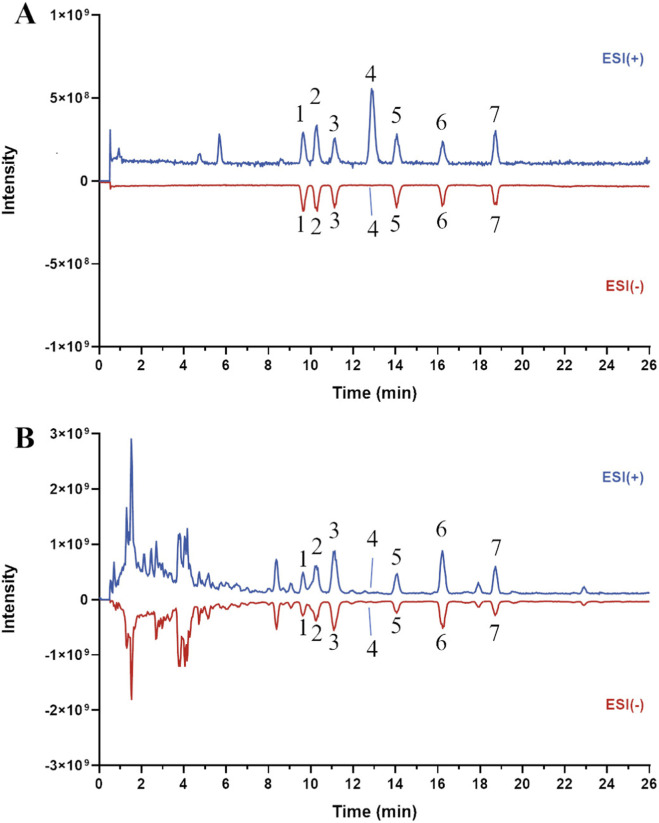
The total ion chromatograms (TICs) of the seven reference substances **(A)** and the extract of TAS **(B)** by UHPLC-Q-Exactive Orbitrap HRMS. 1, astragaloside III; 2, astragaloside IV; 3, astragaloside II; 4, cycloastragenol; 5, isoastragaloside II; 6, astragaloside I; 7, isoastragaloside I.

Astragalus saponins were extracted from *Astragalus membranaceus* (Fisch.) Bge. *var. mongholicus* (Bge.) Hsiao by macroporous resin column chromatography according to the method previously established by our research group (Zhang et al., 2022). *Astragalus membranaceus* (No. 220120) was purchased from Shanghai Hongqiao Traditional Chinese Medicine Decoction Piecess Co., Ltd. (Shanghai, China), and the herb was authenticated as *Astragalus membranaceus* (Fisch.) Bge. var. mongholicus (Bge.) Hsiao by professor LIU Wei, from Shuguang Hospital Affiliated to Shanghai University of Traditional Chinese Medicine. The voucher specimens were deposited in Department of Pharmacy, Shuguang Hospital Affiliated to Shanghai University of Traditional Chinese Medicine. Dried Astragalus membranaceus (2 kg) was cut into segments and extracted three times under reflux with 20L of 70% ethanol (v/v) for 2 h each time. The extracts were combined, filtered, and concentrated under reduced pressure at 45 °C to obtain 4L of concentrated Astragalus extract. The concentrated extract was subjected to separation using macroporous resin column chromatography to prepare saponins (4 kg of concentrate, column volume approximately 3L). A stepwise gradient elution system of water-ethanol (100:0, 70:30, 10:90, v/v) was employed, using 20L for each elution step. Finally, the 90% ethanol fraction (mainly containing saponins) was collected, evaporated under reduced pressure using a rotary evaporator at 45 °C, and the concentrate was freeze-dried to afford the solid material (9.92 g), corresponding to an extract yield of 0.50% from Astragalus. Then, the average content of total Astragalus saponins in the prepared saponins was determined to be 63.38% ± 1.46%. And the chemical analysis of TAS by UHPLC-Q-Orbitrap HRMS (Thermo Fisher Scientific Inc., Grand Island, NY, USA) was described in previous report (Zhang et al., 2024). The total ion chromatograms of the TAS and seven reference standards in positive and negative ion mode are shown in Figure 1A, and the contents of astragaloside I/II/III/IV, cycloastragenol, isoastragaloside I/II are 66.65 mg/g, 56.21 mg/g, 19.12 mg/g, 22.66 mg/g, 0.37 mg/g, 28.38 mg/g and 23.03 mg/g in the TAS, respectively (Figure 1B). Based on preliminary experiments, the oral dose of TAS for rats was set at 40 mg/kg.

### 2.2 Construction of *Numb*-Ex3 overexpression adeno-associated virus vector

*Numb*-Ex3 overexpression (*Numb*-Ex3^OE^) was achieved using a recombinant adeno-associated virus serotype 8 (AAV8). The *Numb*-Ex3 genetic construct was cloned, constructed, and packaged into AAV8 particles (designated *Numb*-Ex3^OE^; titer: 5.41 × 10^13^ v.g./mL; Shanghai Genechem Co., Ltd., China); the target sequence is provided in Supplementary Text 1. For the negative control, an AAV8 empty vector (designated *Numb*-Ex3^EV^; titer: 7 × 10^12^ v.g./mL) from the same manufacturer was used. Each rat received an intrasplenic injection of either the overexpression or empty vector (1.6 × 10^11^ v.g. per rat) concurrently with the BDL surgery.

### 2.3 Reagents

Antibodies used for immunostaining and immunoblotting included: mouse monoclonal against α-smooth muscle actin (α-SMA; ab124964 Abcam, Cambridge, United Kingdom), and hepatocyte nuclear factor 4 alpha (HNF-4α; ab41898, Abcam); rabbit polyclonal against cytokeratin (CK) 7 (155391-1-AP, Proteintech Group Inc., Chicago, IL, USA), CK19 (10712-1-AP, Proteintech), EpCam (ab71916,Abcam), Numb (ab220362,Abcam), RBP-Jκ (ab180588,Abcam), and Hes1 (ab108937, Abcam); and rabbit monoclonal [EPR14335-78] against Sox9 (ab185966,Abcam). A mouse monoclonal antibody against glyceraldehyde-3-phosphate dehydrogenase (GAPDH, Chemicon International, Billerica, MA, USA) served as the loading control. IRDye 800CW donkey anti-mouse IgG (H + L) (LI-COR Bioscience, San Jose, CA, USA) and IRDye 680RD donkey anti-rabbit IgG (H + L) (LI-COR Bioscience).

### 2.4 Animals and experimental protocol

Male Sprague-Dawley (SD) rats (160–180 g) were purchased from Vital River Laboratory Animal Technology Co., Ltd. (Beijing, China). All rats were maintained in ventilated cages under 12-h light/dark cycles with free access to enrichment, water, and feed. All animal experimental protocols were approved by the Animal Research Committee at Shanghai University of Traditional Chinese Medicine (TCM) (PZSHUTCM191122004), and the study protocols adhere to the ARRIVE guideline.

The rat model of CLF was established by BDL as previously described (Xu et al., 2020). Briefly, 42 rats were randomly allocated to a sham-operated group (Sham, *n* = 7) and a model group (*n* = 35). Rats in the model group were anesthetized with sodium pentobarbital (30 mg/kg, i.p.), and a midline laparotomy was performed under aseptic conditions. The common bile duct along with the left and right hepatic ducts were carefully isolated. Ligation was performed on the left/right hepatic ducts and at the hepatic portal and duodenal ends of the common bile duct, after which the abdomen was closed. Sham rats underwent the same surgical procedure except for the ligation step.

Following BDL surgery, the model rats were randomly divided into five groups (*n* = 7 per group): the BDL model group, the AAV8.*Numb*-Ex3 empty vector group (*Numb*-Ex3^EV^), the *Numb*-Ex3^EV^ + TAS (designated TAS) group, the AAV8.*Numb*-Ex3 overexpression group (*Numb*-Ex3^OE^), and the AAV8.*Numb*-Ex3^OE^ + TAS (designated combination therapy) group. Either AAV8.*Numb*-Ex3^OE^ (XM-039111837) or AAV8.*Numb*-Ex3^EV^ (CON462) was administered *via* intrasplenic injection at a dose of 1.6 × 10^11^ viral genomes (v.g.) per rat immediately after BDL. Sham and BDL model group rats received an equivalent volume of physiological saline.

One week after surgery, rats in the TAS-treated groups (*Numb*-Ex3^EV^ + TAS and combination therapy) began receiving a daily oral gavage of TAS (40 mg/kg) for three consecutive weeks. Rats in the remaining control groups received an equal volume of distilled water on the same schedule. All animals had *ad libitum* access to standard laboratory chow throughout the 4-week experimental period. At the endpoint, blood and liver tissue samples were collected for subsequent analysis.

### 2.5 Serum biochemical analysis

Levels of serum biochemical parameters, including alanine aminotransferase (ALT), aspartate aminotransferase (AST), total bilirubin (TBil), direct bilirubin (DBil), alkaline phosphatase (ALP), gamma-glutamyltransferase (GGT), and albumin (Alb) levels, were measured at the Clinical Laboratory Center of Shuguang Hospital affiliated to Shanghai University of TCM.

### 2.6 Histopathological and immunohistochemical analyses

Liver histopathological changes were determined with paraffin-embedded sections stained with hematoxylin and eosin (H&E) or 0.1% (w/v) Sirius Red (Direct Red 80; Aldrich, Milwaukee, WI, USA). Immunohistochemistry was performed was performed on paraffin-embedded sections as previously described (Mu et al., 2010). Briefly, after deparaffinization and rehydration, sections were blocked and then incubated overnight at 4 °C with primary antibodies against α-SMA (1:1,000), CK7 (1:400), CK19 (1:500), HNF4α (1:400), Alb (1:50), and Numb (1:1,000). Subsequently, the sections were incubated with corresponding HRP-conjugated secondary antibodies (1:1,000). Signal visualization was achieved using 3,3′-diaminobenzidine (DAB) substrate with hematoxylin counterstaining. All slides were scanned and imaged using a Leica SCN400 scanner (Leica Microsystems Inc., Concord, ON, Canada).

For immunofluorescence staining, frozen liver tissues were sectioned at a thickness of 6 μm. The sections were used to detect co-expression of the following markers: CK7 (1:200) and OV6 (1:400); CK19 (1:200) and OV6 (1:400); HNF4α (1:1000) and OV6 (1:400). After incubation with primary antibodies and washing, the sections were incubated with Alexa Fluor 488-conjugated goat anti-mouse IgG (A11001; Invitrogen, Carlsbad, CA, USA) and Alexa Fluor 594-conjugated goat anti-rabbit IgG (AB6939; Abcam, Cambridge, United Kingdom). Nuclei were counterstained with 4′,6-diamidino-2-phenylindole (DAPI; 1:1,000). Images were acquired using an FV10i confocal laser scanning microscope (Olympus, Japan).

### 2.7 Measurement of hepatic hydroxyproline content

Hepatic hydroxyproline (Hyp) was measured using a modified method as previously described (Jamall et al., 1981). Briefly, liver tissues were homogenized and hydrolyzed in 6 N HCl at 110 °C for 18 h. The hydrolysate was filtered through a 0.45-μm membrane filter (Millipore, Bedford, MA, USA), followed by the addition of chloramine T to a final concentration of 2.5 mM. The mixture was then treated with 410 mM paradimethylaminobenzaldehyde and incubated at 60 °C for 30 min. After cooling to room temperature, the absorbance was measured at 560 nm using a reagent blank (containing all reagents except tissue) as reference. The Hyp concentration was determined based on a standard curve generated with a commercial Hyp standard (Nakateyitesuku Company, Japan).

### 2.8 Immunoblot analysis

Liver tissue was homogenized in ice-cold RIPA buffer supplemented with protease and phosphatase inhibitors. The homogenates were centrifuged, and the supernatant protein concentration was determined using a BCA assay kit (Thermo Fisher Scientific, USA). Equal amounts of protein (25 µg per lane) were separated by SDS-PAGE and transferred onto PVDF membranes. After blocking with 5% (w/v) bovine serum albumin, the membranes were incubated overnight at 4 °C with the following primary antibodies: α-SMA (1:5,000), CK7 (1:5,000), CK19 (1:1,000), EpCam (1:1,000), Sox9 (1:1,000), HNF4α (1:1,000), Numb (1:1000), RBP-Jκ (1:1,000), Hes1 (1:100), and GAPDH (1:10,000). Subsequently, membranes were incubated with the corresponding secondary antibodies: IRDye 800CW Donkey anti-Mouse IgG (H + L) (1:10,000) and IRDye 680RD Donkey anti-Rabbit IgG (H + L) (1:1,000). Protein bands were visualized using an Odyssey infrared imaging system (LI-COR Biosciences), and band intensity was quantified with Odyssey 2.1 software.

### 2.9 Quantitative real-time PCR analysis

The mRNA expressions of α-SMA, transforming growth factor-beta 1 (TGF-β1), Collagen type I (Col (1)), Col (4), CK7, CK19, EpCam, Sox9, Alb, HNF4α, Numb, Hes1, and RBP-Jκ were quantified by quantitative real-time PCR (qRT-PCR). Total RNA was extracted from frozen liver tissues using Isogen reagent (TOYOBO, Kita-ku, Osaka, Japan). cDNA was synthesized from RNA samples using SuperScript II Reverse Transcriptase (Termo Fisher Scientifc, Waltham, MA, USA). qRT-PCR was performed using SYBR Green Real-Time PCR Master Mix (TOYOBO) according to the manufacturer’s instructions. Sequence-specific primers (listed in Table 1) were designed with Primer Express software (Sigma-Aldrich). Reactions were carried out in triplicate for each sample from seven rats per group. GAPDH was used as the endogenous reference gene. The thermal cycling conditions were as follows: reverse transcription at 42 °C for 15 min, initial denaturation at 95 °C for 2 min, followed by 40 cycles of denaturation at 95 °C for 15 s, and annealing/extension at 60 °C for 1 min.

**TABLE 1:** Primer pairs and probes used for real-time PCR.

Primer name	Primer sequence (5′→3′)	Note
α-SMA	Forward AATGGCTCTGGGCTCTGTAA Reverse TCTCTTGCTCTGGGCTTCAT	SYBR Green
TGF-β1	Forward ATTCCTGGCGTTACCTTGG Reverse AGCCCTGTATTCCGTCTCCT	SYBR Green
Col1	Forward ACGTCCTGGTGAAGTTGGTC Reverse TCCAGCAATACCCTGAGGTC	SYBR Green
Col4	Forward TTCCAGGGTTACAAGGTGT Reverse AGTCCAGGTTCTCCAGCATC	SYBR Green
CK7	Forward AGGAACAGAAGTCAGCCAAGAG Reverse GCAACACAAACTCATTCTCAGC	SYBR Green
CK19	Forward GATCTGCGTAGTGTGG Reverse AAAACCAAACTGGGGATG	SYBR Green
EpCam	Forward ACTTGATGTGGATGTGTGCGT Reverse AAGGTCGTGCCGATGTCTCTC	SYBR Green
Sox9	Forward GAAAGACCACCCCGATTACAAG Reverse AAGATGGCGTTAGGAGAGATGTG	SYBR Green
ALB	Forward CTATCTGTCTGCCATCCTGAA Reverse CACTACAGCACTTGGTGACCT	SYBR Green
HNF4α	Forward CTATCTGTCTGCCATCCTGAA Reverse CACTACAGCACTTGGTGACCT	SYBR Green
Numb	Forward GCTACTTTCGATGCCAGTAGAACCA Reverse CTGTTGCCAGGAGCCACTGA	SYBR Green
Hes1	Forward GACGGCCAATTTGCTTTC Reverse GACACTGCGTTAGGACCC	SYBR Green
RBP-Jκ	Forward TTGCTTACCTTCAGGCGTGTG Reverse GCCCAATGAGTCTGCTGCAA	SYBR Green
GAPDH	Forward AAGGTCATCCATGACAACTTTGGC Reverse ACAGTCTTCTGGGTGGCAGTGAT	SYBR Green

### 2.10 *In vitro* experimental protocol

*In vitro* studies were performed using the WB-F344 cell line, a well-characterized rat hepatic progenitor cell model that retains key morphological and functional features of primary liver progenitor cells (Tsao et al., 1984).

WB-F344 cells were maintained in Dulbecco’s Modified Eagle Medium (DMEM; Life Technologies) supplemented with 10% fetal bovine serum (Gibco). To induce differentiation, cells were seeded onto six-well Permanox Lab-Tek chamber slides (Nalge Nunc International, Naperville, IL, USA) at a density of 2 × 10^3^ cells/cm^2^. After 24 h, cells were divided into the following treatment groups (*n* = 3 per group): normal control (N), Sodium butyrate (SB; 3.75 mM; Sigma, B5887-1G) only, SB + Lentiviral (LV).*Numb*-Ex3 empty vector (*Numb*-Ex3^EV^), SB + *Numb*-Ex3^EV^ + TAS (TAS), SB + LV.*Numb*-Ex3^OE^ (*Numb*-Ex3^OE^), and SB + LV-*Numb*-Ex3^OE^ + TAS (*Numb*-Ex3^OE^ + TAS). LV transduction with LV-*Numb*-Ex3^OE^ or control vector was performed at a multiplicity of infection (MOI) of 50; the corresponding target sequences are provided in Supplementary Text 2 and Text 3, respectively. At 6 h post-transduction, the medium was replaced with fresh medium containing SB, with or without TAS as indicated. The medium was refreshed every 48 h, and the total culture duration was 7 days.

### 2.11 Statistical analysis

All quantitative data are presented as Mean ± SD. Statistical comparisons across multiple groups were performed using one-way analysis of variance (ANOVA), followed by Tukey’s *post hoc* test for inter-group comparisons, using SPSS 24.0 software. A difference was considered statistically significant at *P* < 0.05.

## 3 Results

### 3.1 Combined TAS and *Numb*-Ex3^OE^ treatment produces synergistic anti-fibrotic effects in CLF

**FIGURE 2:**
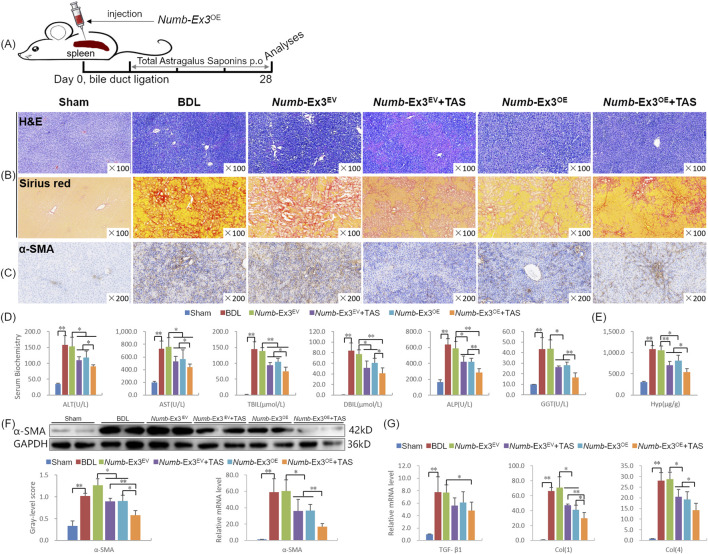
TAS Potentiates Numb-Exon3^OE^-Mediated Protection Against Liver Fibrosis. **(A)** Experimental design schematic; **(B)** H&E staining (×100) and Sirius red collagen staining (×100); **(C)** α-SMA Immunohistochemical staining (×200) **(D)** Serum levels of biochemical markers; **(E)** Hyp content in liver tissue; **(F)** α-SMA immunoblotting bands, gray-level integrations and mRNA expression levels; **(G)** TGF-β1, Col (1), and Col (4) mRNA expression. Ex3, Exon3. *P < 0.05, **P < 0.01.

To evaluate whether the combination of TAS and *Numb*-Ex3^OE^ could enhance anti-fibrotic efficacy, rats received an intra-splenic injection of AAV8.*Numb*-Ex3^OE^ immediately after BDL surgery, followed by oral administration of TAS beginning at week 2. Tissue samples were collected at week 4 for analysis (Figure 2A). Histopathological examination showed that the combination therapy synergistically attenuated hepatic inflammation, bile duct hyperplasia, and collagen deposition more effectively than either monotherapy (Figure 2B). Serum biochemical assays indicated that both TAS and *Numb*-Ex3^OE^ significantly reduced the activities of ALT, AST, ALP, GGT, as well as the levels of TBil and DBil (*P* < 0.05 or *P* < 0.01). Notably, the combination therapy group exhibited further decreases in these parameters compared to the monotherapy groups (*P* < 0.05 or *P* < 0.01), with ALP and GGT showing the most pronounced reductions (Figure 2D). Hepatic Hyp content was also significantly reduced by both treatments individually (*P* < 0.05 or *P* < 0.01), while the combination therapy resulted in additional reductions of 22.9% and 33.5% compared to TAS and *Numb*-Ex3^OE^ groups, respectively (*P* < 0.05; Figure 2E). These results demonstrate that the combination of TAS and *Numb*-Ex3^OE^ exerts a synergistic therapeutic effect on CLF.

Since HSC activation is a key driver of liver fibrosis, with α-SMA serving as a marker for HSCs activation (Yin et al., 2013), we evaluated α-SMA expression by immunostaining and immunoblotting. α-SMA-positive area was significantly reduced in both the TAS and *Numb*-Ex3^OE^ groups compared to the *Numb*-Ex3^EV^ group, and further decreased in the combination group (Figure 2C). Consistent with immunostaining, Western blot analysis revealed that α-SMA protein expression was reduced by 29.4% and 28.5% in the TAS and *Numb*-Ex3^OE^ groups, respectively (*P* < 0.05 vs. *Numb*-Ex3^EV^), while the combination therapy led to additional reductions of 36.8% and 37.5% compared to the corresponding monotherapies (*P* < 0.05, *P* < 0.01). A similar suppression pattern was observed at the mRNA level (Figure 2F). Moreover, mRNA expression of Col (1) and Col (4) was significantly lower in the combination therapy group than in either monotherapy group (*P* < 0.05 or *P* < 0.01; Figure 2G). These data suggest that the synergistic anti-fibrotic effect of TAS and *Numb*-Ex3^OE^ is mediated, at least in part, through enhanced suppression of HSC activation.

### 3.2 Combination TAS and *Numb*-Ex3^OE^ synergistically suppress ductular reaction derived from hepatic progenitor cells in CLF

**FIGURE 3:**
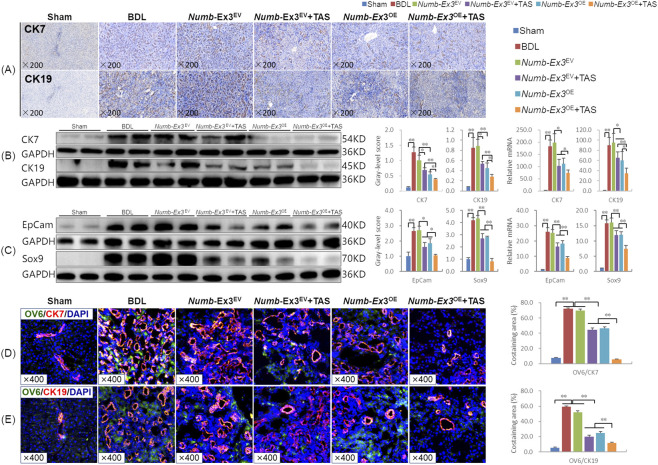
TAS combined with Numb-Exon3^OE^ synergistically suppression of hepatic progenitor cell activation and ductular reaction **(A)** CK7 and CK19 immunohistochemical staining (×200). **(B, C)** CK7, CK19, EpCAM, and Sox9 immunoblotting bands, gray-level integrations; **(D, E)** OV6/CK7, OV6/CK19 immunofluorescence co-staining and the co-staining area ratio (The presented animal experiments were conducted in the same batch, and the sham group, BDL model group, Numb-Ex3EV group, and Numb-Ex3^OE^ group used the same images as those in the published article (Xu et al., 2025), licensed under CC BY, and are reproduced here with permission). Ex3, Exon3. *P < 0.05, **P < 0.01.

DR driven by Notch signaling is a key pathogenic feature of CLF (Zhang et al., 2016). Immunostaining demonstrated showed that positive staining for the biliary markers CK7 and CK19 were markedly reduced in the TAS, *Numb*-Ex3^OE^, and combination therapy groups, with the strongest suppression observed in the combination therapy group (Figure 3A). Corroborating these findings, the combination therapy decreased CK7 protein expression by 43.7% and 27.9% (*P* < 0.05 or *P* < 0.01), and CK19 expression by 47.9% and 37.4% (*P* < 0.05 or *P* < 0.01), compared to TAS and *Numb*-Ex3^OE^ monotherapies, respectively. Corresponding decreases in CK7 and CK19 mRNA levels were also detected (Figure 3B), indicating that combination therapy synergistically suppresses DR.

Since HPCs are the primary cellular source of DR in CLF (Zhang et al., 2016), we next examined the expression of HPC markers. The combination therapy significantly downregulated the protein levels of EpCAM and Sox9. Specifically, EpCAM expression was reduced by 35.0% and 43.5% (*P* < 0.05), and Sox9 by 69.2% and 70.5% (*P* < 0.01), relative to TAS and *Numb*-Ex3^OE^ monotherapies, respectively. These reductions were also observed at the mRNA level (Figure 3C). Immunofluorescence co-staining further demonstrated extensive OV6/CK7 and OV6/CK19 co-expression in BDL and *Numb*-Ex3^EV^ groups. Both TAS and *Numb*-Ex3^OE^ monotherapies significantly reduced these dual-positive areas compared to the BDL group (*P* < 0.05), while, the combination therapy led to a further marked decrease relative to either monotherapies (*P* < 0.01) (Figures 3D,E). Together, these results demonstrate that the combination of TAS and *Numb*-Ex3^OE^ synergistically inhibits HPC differentiation into cholangiocytes, highlighting its enhanced therapeutic potential in CLF.

### 3.3 Combined TAS and *Numb*-Ex3^OE^ treatment synergistically promotes hepatocyte proliferation

**FIGURE 4:**
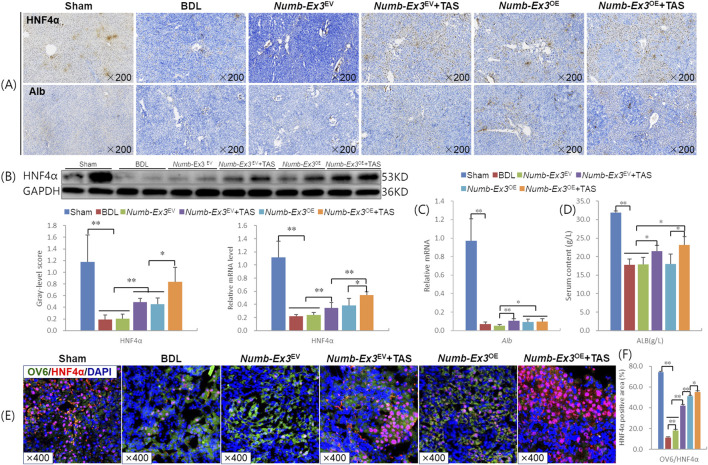
Synergistic promotion of hepatocyte proliferation and Alb synthesis by combination therapy **(A)** HNF4α, Alb immunohistochemical staining (×200); **(B)** HNF4α immunoblotting bands, gray-level integrations and mRNA expression levels; **(C)** Alb mRNA expression level and **(D)** Serum Alb content. **(E)** HNF4α immunofluorescence staining and **(F)** the staining area ratio. Ex3, Exon3. *P < 0.05, **P < 0.01.

HNF4α and Alb are well-established markers of mature hepatocytes (Mann et al., 2023; Aizarani et al., 2019). Immunostaining showed markedly reduced HNF4α^+^ and Alb^+^ cells in the BDL and *Numb*-Ex3^EV^ groups, whereas all treatment groups exhibited increased positivity, with the most pronounced enhancement observed in the combination therapy group (Figure 4A). Western blot analysis demonstrated that both *Numb*-Ex3^OE^ and TAS monotherapies significantly upregulated hepatic HNF4α protein and mRNA expression compared to BDL and *Numb*-Ex3^EV^ controls (*P* < 0.01). Notably, the combination therapy further enhanced HNF4α levels versus either monotherapy (*P* < 0.05 or *P* < 0.01) (Figure 4B). Although Alb mRNA expression was significantly elevated in the combination therapy group versus *Numb*-Ex3^EV^ (Figure 4C), serum Alb content was significant higher versus BDL, *Numb*-Ex3^EV^, and *Numb*-Ex3^OE^ groups (*P* < 0.05), and showed a non-significant upward trend versus TAS monotherapy (*P* = 0.33) (Figure 4D). Immunofluorescence confirmed that the HNF4α expressed area was increased significantly in the combination therapy group than either monotherapy (*P* < 0.01) (Figures 4E, F). These findings collectively indicate that combination therapy synergistically promote hepatocyte proliferation and albumin synthesis.

### 3.4 Combined TAS and *Numb*-Ex3^OE^ treatment synergistically inhibits the notch signaling activation

**FIGURE 5:**
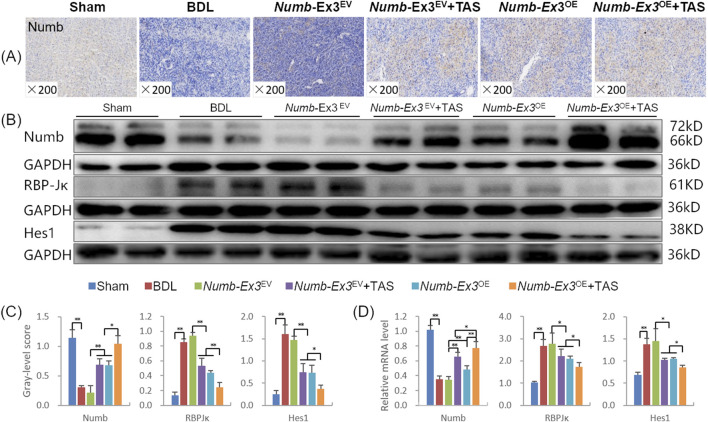
The combination therapy of TAS and Numb-Exon3^OE^ synergistically inhibit notch signaling activation **(A)** Numb immunohistochemical staining (×200). **(B)** Numb, RBP-Jκ, and Hes1 immunoblotting bands. **(C)** Numb, RBP-Jκ, and Hes1 gray-level integrations. **(D)** Numb, RBP-Jκ, and Hes1 mRNA expression levels. Ex3, Exon3. *P < 0.05, **P < 0.01.

Activation of Notch signaling is a key pathogenic mechanism in CLF, and blocking this pathway effectively inhibits CLF progression (Zhang et al., 2016). To investigate the impact of hepatic *Numb*-Ex3 on Notch signaling activation, we measured the expression levels of Numb, its downstream transcription factor RBP-Jκ (a Notch signaling transcription factor), and the Notch signaling target gene Hes1. Immunostaining showed that markedly reduced Numb-positive staining in the BDL and Numb-Exon3^EV^ groups, while all intervention groups exhibited markedly increased Numb positivity (Figure 5A). Western blot analysis revealed that both *Numb*-Ex3^OE^ and TAS significantly increased hepatic Numb protein expression, while decreasing RBP-Jκ and Hes1 protein levels (*P* < 0.01), indicating their inhibitory effects on Notch signaling activation. Notably, the combination therapy further enhanced these effects: Numb protein expression increased by 52.0% and 53.5% compared to the TAS and *Numb*-Ex3^OE^ groups, respectively (*P* < 0.05); RBP-Jκ protein expression decreased by 55.1% and 45.5% (*P* < 0.01); and Hes1 levels declined by 51.2% and 50.0% (*P* < 0.05) (Figures 5B,C). The mRNA expression patterns of Numb, RBP-Jκ, and Hes1 were consistent with their protein expression trends (Figure 5D). These findings demonstrate that the combination therapy synergistically suppresses Notch signaling pathway activation.

### 3.5 Combined TAS and *Numb*-Ex3^OE^ synergistically regulates the differentiation fate of HPCs *in vitro*

**FIGURE 6:**
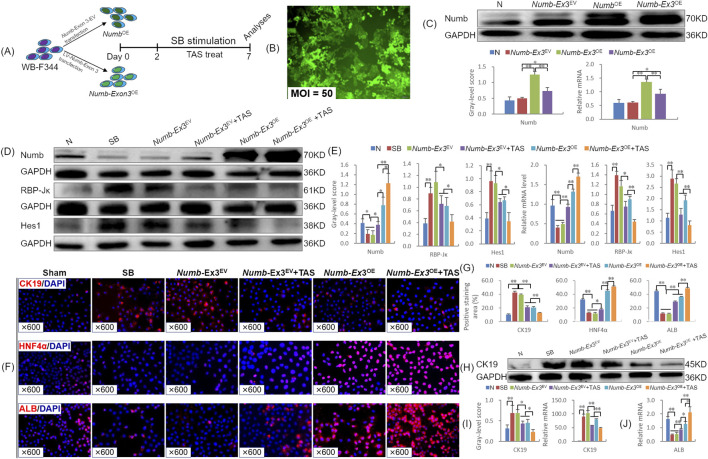
Combination therapy of TAS and Numb-Exon3^OE^ synergistically regulate the differentiation fate of WB-F344 cells *in vitro* **(A)** Experimental flow chart; **(B)** Cell morphology and EGFP expression after lentivirus transfection for 72 h (× 100); **(C)** Verification of successful overexpression of Numb and Numb-Exon3 through Numb protein and mRNA expression (The presented *in vitro* experiments were all conducted in the same batch. The Numb protein blot images used are the same as those used in the published article (Xu et al., 2025), licensed under CC BY, and are reproduced here with permission); **(D)** Numb, RBP_Jκ, and Hes1 immunoblotting bands, and **(E)** gray-level integrations and mRNA expression levels (n = 3/per group); **(F)** CK19, HNF4α and Alb immunofluorescence staining (× 600), and **(G)** positive area of CK19, HNF4α and Alb (%); **(H)** CK19 immunoblotting bands, and **(I)** gray-level integrations and mRNA expression level of CK19 (n = 3/per group); **(J)** Alb mRNA expression level (n = 3/per group). Ex3, Exon3. *P < 0.05, **P < 0.01.

To investigate the regulatory effects of TAS, *Numb*-Ex3, and their combination on HPCs differentiation, we overexpressed *Numb*-Ex3 (*Numb*-Ex3^OE^) in WB-F344 cells and induced differentiation using sodium butyrate (SB), as outlined in Figure 6A. LV transfection at an MOI of 50 achieved an efficiency exceeding 80%, with no adverse effects on cell morphology (Figure 6B). Both *Numb* ^OE^ (positive control) and *Numb*-Ex3^OE^ groups increased significantly Numb protein expression compared to the *Numb*-Ex3^EV^ group (*P* < 0.05 or *P* < 0.01), and mRNA expression trends were consistent (Figure 6C), confirming successful model establishment.

Based on CCK-8 assays, a TAS concentration of 100 μg/mL (selected from a range of 25–100 μg/mL) was identified as optimal for promoting WB-F344 cell proliferation (*P* < 0.05 or *P* < 0.01; Supplementary Figure S1). Western blot analysis showed that TAS and *Numb*-Ex3^OE^ monotherapies increased Numb protein expression by 1.2-fold and 3.5-fold, respectively, relative to the *Numb*-Ex3^EV^ group (*P* < 0.05 or *P* < 0.01). The combination therapy further enhanced Numb expression, resulting in a 2.3-fold increase over TAS alone and a 57.7% increase over *Numb*-Ex3^OE^ alone, representing a 1.9-fold elevation compared to controls (*P* < 0.05 or *P* < 0.01). Corresponding changes were observed at the mRNA level (Figures 6D,E). In parallel, both monotherapies suppressed the expression of RBP-Jκ and Hes1 (protein and mRNA), with the strongest inhibition seen in the combination group (*P* < 0.05 or *P* < 0.01), indicating a synergistic blockade of the Notch pathway.

To evaluate differentiation outcomes, we analyzed the cholangiocyte marker CK19 and the hepatocyte markers HNF4α and Alb. Immunostaining showed that SB-induced differentiation in the presence of the *Numb*-Ex3^EV^ led to an increased CK19-positive area (*P* < 0.01) and decreased HNF4α- and Alb-positive areas (*P* < 0.01). These effects were reversed by TAS or *Numb*-Ex3^OE^ monotherapy, which reduced CK19 and increased HNF4α and Alb expression (*P* < 0.01). The combination therapy produced additive effects, further enhancing the expression of HNF4α and Alb, while suppressing the CK19 (Figures 6F,G). These findings were corroborated by protein and mRNA levels of CK19 (Figures 6H,I) and Alb mRNA expression (Figure 6J). These results indicated that the combination of TAS and *Numb*-Ex3^OE^ not only inhibits the differentiation HPCs into cholangiocytes, thereby suppressing DR; but also promotes the differentiation HPCs into hepatocytes, highlighting its synergistic role in regulating HPCs fate *in vitro*.

## 4 Discussion

Numb, an evolutionarily conserved gene first identified in *Drosophila* sensory organ precursor cells, acts as a negative regulator of the Notch signaling pathway by antagonizing Notch family membrane receptors through asymmetric mitosis to govern stem cell fate (Colaluca et al., 2018; Uemura et al., 1989). In human hepatocellular carcinoma, Numb expression is significantly downregulated, and its restoration *via* miR-148a-mediated mechanisms inhibits Notch signaling and suppresses tumor progression (Jung et al., 2016). However, the therapeutic potential of Numb in CLF remains unexplored. Our previous studies revealed a marked reduction of Numb in both PBC patients and BDL rat models (Xu et al., 2023). Furthermore, AAV8.*Numb* supplementation in rat livers exhibited potent against CLF effects (Xu et al., 2022).

In mammals, alternative splicing of Ex3 and 9 in the Numb gene gives rise to two principal functional domains: the PTB domain and the proline-rich region (PRR). Based on the inclusion or exclusion of an 11-amino acid segment (encoded by Ex3) in the PTB domain and a 48-amino acid segment (encoded by Ex9) in the PRR, Numb proteins are categorized into four main isoforms (Confalonieri et al., 2019).

Recent studies have revealed functional differences among Numb isoforms. For instance, research has shown that increased expression of Numb isoforms containing Ex9 paradoxically promotes Notch signaling activation (Zong et al., 2014; Bechara et al., 2013). In lung cancer, Numb2 (PTB_L_/PRR_S_) was shown to inhibit Notch signaling, but this suppression was counteracted by Numb1 (PTB_L_/PRR_L_)—which contains the Ex9-encoded PRR—leading to increased expression of Notch target genes (Misquitta-Ali et al., 2011). In contrast, isoforms harboring an intact Ex3-encoded PTB domain recruit E3 ubiquitin ligases such as LNX and ITCH to promote ubiquitination and subsequent proteasomal degradation of Notch receptors, thereby attenuating Notch signaling (Nie et al., 2004; McGill and McGlade, 2003).

These findings collectively suggest that Ex3 constitutes a critical determinant for the negative regulation of Notch by Numb, and that Ex3-containing isoforms may play a particularly important role in mediating the against CLF effects of Numb. However, the relationship between these specific Numb isoforms and HPCs differentiation remains poorly understood and requires further investigation.

TAS, the primary active components extracted from *Astragalus membranaceus,* exhibit diverse pharmacological activities including immunomodulation, multi-organ protection, and antioxidant, antiviral, and antitumor effects (Guo et al., 2019; Ma et al., 2023). Our previous research indicated that TAS alleviates CLF by inhibiting Notch signaling activation in HPCs, suppressing their differentiation into cholangiocytes, and upregulating Numb expression (Yongping et al., 2015). Based on these findings, the current study investigated the combined effect of TAS and *Numb*-Ex3 overexpression in a rat model of BDL-induced CLF.

*In vivo* results demonstrated that, compared to TAS or *Numb*-Ex3^OE^ alone, the combination therapy further improved serum biochemical parameters, particularly those associated with cholestasis including TBil, ALP, and γ-GT in BDL rats. Moreover, the combination therapy more effectively attenuated collagen fiber deposition than either treatment alone, evidenced by decreased hepatic Hyp content and downregulated mRNA expression of Col (1) and Col (4). It also resulted in a more pronounced reduction in α-SMA expression, indicating stronger suppression of HSC activation.

To assess HPCs differentiation, we examined markers of HPCs, BECs, and hepatocytes. The combination therapy more potently inhibited HPCs differentiation into BECs, as shown by reduced expression of EpCam, Sox9, CK7, and CK19, compared to either monotherapy. In contrast, it promoted hepatocyte proliferation, reflected by increased expression of the hepatocyte-specific markers HNF4α and Alb (Kotulkar et al., 2023; Chen et al., 2012). *In vitro* experiments corroborated these findings, showing that TAS combined with *Numb*-Ex3^OE^ further decreased CK19 expression while increasing Alb levels. Together, these findings support synergistic against CLF effects of the combination treatment.

We further evaluated the influence of the combined therapy on the Notch pathway. Compared to monotherapy, the combined therapy exhibited further elevated Numb expression, along with markedly reduced levels of the downstream Notch effectors RBP-Jκ and Hes1, indicating enhanced suppression of Notch signaling. *In vitro* experiments on DR yielded consistent results. Collectively, these data confirm that TAS inhibits CLF progression, in line with our previous reports, and that its combination with *Numb*-Ex3^OE^ further upregulates hepatic Numb expression and more strongly inhibits Notch signaling activation. The synergistic against CLF effect is underpinned by suppressed DR and enhanced hepatocyte proliferation, orchestrated through the regulated fate of HPCs.

However, as *Numb*-Ex3 knockout studies have not yet been performed, it remains unclear whether TAS exerts it’s against CLF effects specifically through *Numb*-Ex3–mediated inhibition of Notch signaling. Therefore, future studies will involve generating *Numb*-Ex3 knockout mouse models to further elucidate the role of this isoform in regulating HPC differentiation and its functional interplay with TAS.

## 5 Conclusion

This study demonstrates that combined therapy with TAS and *Numb*-Ex3 overexpression exerts superior anti-cholestatic fibrotic effects compared to either treatment alone. The underlying mechanism involves suppression of Notch signaling pathway activation, thereby inhibiting the activation of DR and HSCs, while enhancing the hepatocytic differentiation of HPCs. Our findings posit that a combinatorial approach integrating a TCM extract with molecular targeted-gene therapy offers a promising novel strategy for treating cholestatic fibrosis.

## Data Availability

The original contributions presented in the study are included in the article/Supplementary Material, further inquiries can be directed to the corresponding authors.

## References

[B1] Aizarani N. Saviano A. Sagar, Mailly L. Durand S. Herman J. S. (2019). A human liver cell atlas reveals heterogeneity and epithelial progenitors. Nature 572 (7768), 199–204. 10.1038/s41586-019-1373-2 31292543 PMC6687507

[B2] Bechara E. G. Sebestyén E. Bernardis I. Eyras E. Valcárcel J. (2013). RBM5, 6, and 10 differentially regulate NUMB alternative splicing to control cancer cell proliferation. Mol. Cell. 52 (5), 720–733. 10.1016/j.molcel.2013.11.010 24332178

[B3] Chen Y. F. Tseng C. Y. Wang H. W. Kuo H. C. Yang V. W. Lee O. K. (2012). Rapid generation of mature hepatocyte-like cells from human induced pluripotent stem cells by an efficient three-step protocol. Hepatology 55 (4), 1193–1203. 10.1002/hep.24790 22095466 PMC3779307

[B4] Colaluca I. N. Tosoni D. Nuciforo P. Senic-Matuglia F. Galimberti V. Viale G. (2008). NUMB controls p53 tumour suppressor activity. Nature 451 (7174), 76–80. 10.1038/nature06412 18172499

[B5] Colaluca I. N. Basile A. Freiburger L. D'Uva V. Disalvatore D. Vecchi M. (2018). A Numb-Mdm2 fuzzy complex reveals an isoform-specific involvement of numb in breast cancer. J. Cell. Biol. 217 (2), 745–762. 10.1083/jcb.201709092 29269425 PMC5800818

[B6] Confalonieri S. Colaluca I. N. Basile A. Pece S. Di Fiore P. P. (2019). Exon 3 of the NUMB gene emerged in the chordate lineage coopting the NUMB protein to the regulation of MDM2. G3 (Bethesda) 9 (10), 3359–3367. 10.1534/g3.119.400494 31451549 PMC6778778

[B7] Corpechot C. Carrat F. Bonnand A. M. Poupon R. E. Poupon R. (2000). The effect of ursodeoxycholic acid therapy on liver fibrosis progression in primary biliary cirrhosis. Hepatology 32 (6), 1196–1199. 10.1053/jhep.2000.20240 11093724

[B8] Fabris L. Fiorotto R. Spirli C. Cadamuro M. Mariotti V. Perugorria M. J. (2019). Pathobiology of inherited biliary diseases: a roadmap to understand acquired liver diseases. Nat. Reviews. Gastroenterol. Hepatol. 16 (8), 497–511. 10.1038/s41575-019-0156-4 31165788 PMC6661007

[B9] Guo Z. Lou Y. Kong M. Luo Q. Liu Z. Wu J. (2019). A systematic review of phytochemistry, pharmacology and pharmacokinetics on astragali radix: implications for astragali radix as a personalized medicine. Int. J. Mol. Sci. 20 (6), 1463. 10.3390/ijms20061463 30909474 PMC6470777

[B10] Jamall I. S. Finelli V. N. Que Hee S. S. (1981). A simple method to determine nanogram levels of 4-hydroxyproline in biological tissues. Anal. Biochem. 112 (1), 70–75. 10.1016/0003-2697(81)90261-x 7258630

[B11] Jung K. H. Zhang J. Zhou C. Shen H. Gagea M. Rodriguez-Aguayo C. (2016). Differentiation therapy for hepatocellular carcinoma: multifaceted effects of miR-148a on tumor growth and phenotype and liver fibrosis. Hepatology 63 (3), 864–879. 10.1002/hep.28367 26599259 PMC4764447

[B12] Kim H. Ronai Z. A. (2018). Rewired Notch/p53 by numb'ing Mdm2. J. Cell. Biol. 217 (2), 445–446. 10.1083/jcb.201712007 29339436 PMC5800819

[B13] Kotulkar M. Robarts D. R. Apte U. (2023). HNF4α in hepatocyte health and disease. Semin. Liver Dis. 43 (2), 234–244. 10.1055/a-2097-0660 37216979 PMC10947958

[B14] Koyama Y. Brenner D. A. (2017). Liver inflammation and fibrosis. J. Clin. Investig. 127 (1), 55–64. 10.1172/JCI88881 28045404 PMC5199698

[B15] Loeffler D. Wehling A. Schneiter F. Zhang Y. Müller-Bötticher N. Hoppe P. S. (2019). Asymmetric lysosome inheritance predicts activation of haematopoietic stem cells. Nature 573 (7774), 426–429. 10.1038/s41586-019-1531-6 31485073

[B16] Ma L. La X. Zhang B. Xu W. Tian C. Fu Q. (2023). Total astragalus saponins can reverse type 2 diabetes mellitus-related intestinal dysbiosis and hepatic insulin resistance *in vivo* . Front. Endocrinol. 14, 1190827. 10.3389/fendo.2023.1190827 38053727 PMC10694298

[B17] Machida K. (2020). Cell fate, metabolic reprogramming and lncRNA of tumor-initiating stem-like cells induced by alcohol. Chem. Biol. Interact. 323, 109055. 10.1016/j.cbi.2020.109055 32171851 PMC7238551

[B18] Mann J. P. Lenz D. Stamataki Z. Kelly D. (2023). Common mechanisms in pediatric acute liver failure. Trends Mol. Med. 29 (3), 228–240. 10.1016/j.molmed.2022.11.006 36496278

[B19] Mayo M. J. (2022). Mechanisms and molecules: what are the treatment targets for primary biliary cholangitis? Hepatology 76 (2), 518–531. 10.1002/hep.32405 35152430

[B20] McGill M. A. McGlade C. J. (2003). Mammalian numb proteins promote Notch1 receptor ubiquitination and degradation of the Notch1 intracellular domain. J. Biol. Chem. 278 (25), 23196–23203. 10.1074/jbc.M302827200 12682059

[B21] Misquitta-Ali C. M. Cheng E. O'Hanlon D. Liu N. McGlade C. J. Tsao M. S. (2011). Global profiling and molecular characterization of alternative splicing events misregulated in lung cancer. Mol. Cell. Biol. 31 (1), 138–150. 10.1128/MCB.00709-10 21041478 PMC3019846

[B22] Mu Y. P. Ogawa T. Kawada N. (2010). Reversibility of fibrosis, inflammation, and endoplasmic reticulum stress in the liver of rats fed a methionine-choline-deficient diet. Lab. Investig. 90 (2), 245–256. 10.1038/labinvest.2009.123 19949375

[B23] Nevens F. Andreone P. Mazzella G. Strasser S. I. Bowlus C. Invernizzi P. (2016). A placebo-controlled trial of obeticholic acid in primary biliary cholangitis. N. Engl. J. Med. 375 (7), 631–643. 10.1056/NEJMoa1509840 27532829

[B24] Nie J. Li S. S. McGlade C. J. (2004). A novel PTB-PDZ domain interaction mediates isoform-specific ubiquitylation of Mammalian numb. J. Biol. Chem. 279 (20), 20807–20815. 10.1074/jbc.M311396200 14990566

[B25] Shu Y. Xu Q. Xu Y. Tao Q. Shao M. Cao X. (2021). Loss of numb promotes hepatic progenitor expansion and intrahepatic cholangiocarcinoma by enhancing notch signaling. Cell. Death Dis. 12 (11), 966. 10.1038/s41419-021-04263-w 34667161 PMC8526591

[B26] Su D. Li Y. Zhang W. Gao H. Cheng Y. Hou Y. (2023). SPTAN1/NUMB axis senses cell density to restrain cell growth and oncogenesis through hippo signaling. J. Clin. Investig. 133 (20), e168888. 10.1172/JCI168888 37843276 PMC10575737

[B27] Tsao M. S. Smith J. D. Nelson K. G. Grisham J. W. (1984). A diploid epithelial cell line from normal adult rat liver with phenotypic properties of 'oval' cells. Exp. Cell. Res. 154 (1), 38–52. 10.1016/0014-4827(84)90666-9 6468534

[B28] Uemura T. Shepherd S. Ackerman L. Jan L. Y. Jan Y. N. (1989). Numb, a gene required in determination of cell fate during sensory organ formation in drosophila embryos. Cell. 58 (2), 349–360. 10.1016/0092-8674(89)90849-0 2752427

[B29] Xu W. Xu Y. N. Zhang X. Xu Y. Jian X. Chen J. M. (2020). Hepatic stem cell numb gene is a potential target of huang Qi decoction against cholestatic liver fibrosis. Sci. Rep. 10 (1), 17486. 10.1038/s41598-020-74324-1 33060633 PMC7566460

[B30] Xu Y. N. Xu W. Chen J. M. Liu W. Chen G. F. Zhang H. (2022). Overexpression of numb gene can inhibit the progression of biliary liver fibrosis in adult liver. Zhonghua Gan Zang Bing Za Zhi 30 (11), 1175–1181. 10.3760/cma.j.cn501113-20210222-00091 36891694 PMC12770771

[B31] Xu Y. N. Xu W. Zhang X. Wang D. Y. Zheng X. R. Liu W. (2023). BM-MSCs overexpressing the numb enhance the therapeutic effect on cholestatic liver fibrosis by inhibiting the ductular reaction. Stem Cell. Res. Ther. 14 (1), 45. 10.1186/s13287-023-03276-w 36941658 PMC10029310

[B38] Xu Y. N. Zong M. Y. Xu W. Zhang S. H. Wang D. Y. Zheng X. R. (2025). Numb-exon3 and full length Numb equivalently alleviate cholestatic liver fibrosis by inhibiting ductular reaction. Sci. Rep. 15 (1), 39983. 10.1038/s41598-025-23696-3 41238697 PMC12618559

[B32] Yin C. Evason K. J. Asahina K. Stainier D. Y. (2013). Hepatic stellate cells in liver development, regeneration, and cancer. J. Clin. Investig. 123 (5), 1902–1910. 10.1172/JCI66369 23635788 PMC3635734

[B33] Yongping M. Zhang X. Xuewei L. Fan W. Chen J. Zhang H. (2015). Astragaloside prevents BDL-Induced liver fibrosis through inhibition of notch signaling activation. J. Ethnopharmacol. 169, 200–209. 10.1016/j.jep.2015.04.015 25917841

[B34] Zhang X. Du G. Xu Y. Li X. Fan W. Chen J. (2016). Inhibition of notch signaling pathway prevents cholestatic liver fibrosis by decreasing the differentiation of hepatic progenitor cells into cholangiocytes. Lab. Investig. 96 (3), 350–360. 10.1038/labinvest.2015.149 26692291

[B35] Zhang L. Hu Y. Qi S. Zhang C. Zhou Q. Zhang D. (2022). Astragalus saponins and its main constituents ameliorate ductular reaction and liver fibrosis in a mouse model of DDC-Induced cholestatic liver disease. Front. Pharmacol. 13, 965914. 10.3389/fphar.2022.965914 36339578 PMC9632275

[B36] Zhang L. Shi J. Shen Q. Fu Y. Qi S. Wu J. (2024). Astragalus saponins protect against extrahepatic and intrahepatic cholestatic liver fibrosis models by activation of farnesoid X receptor. J. Ethnopharmacol. 318 (Pt A), 116833. 10.1016/j.jep.2023.116833 37400008

[B37] Zong F. Y. Fu X. Wei W. J. Luo Y. G. Heiner M. Cao L. J. (2014). The RNA-Binding protein QKI suppresses cancer-associated aberrant splicing. PLoS Genet. 10 (4), e1004289. 10.1371/journal.pgen.1004289 24722255 PMC3983035

