# Supplementary material for: Synergistic amelioration of cholestatic liver fibrosis by combined total astragalus saponins and AAV8.Numb-Exon3 through modulation of hepatic progenitor cell differentiation
Source: Front Pharmacol. 2026 Apr 23;17:1762397. doi: 10.3389/fphar.2026.1762397 (PMC13149365; doi:10.3389/fphar.2026.1762397)

Supplementary Materials

**Figure S1**

**
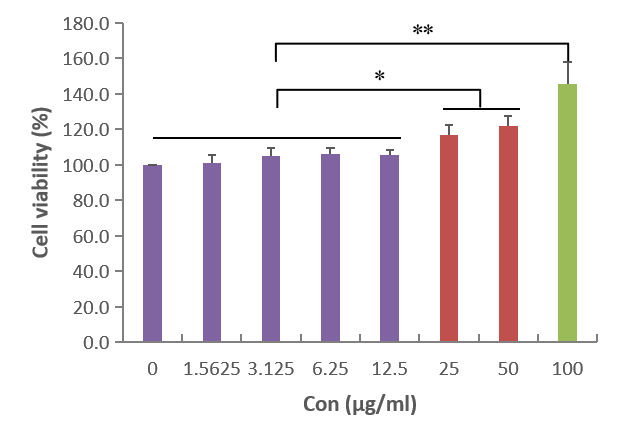
**

**Suppl. Fig. 1. Optimal Drug Concentration Screening for TAS:**

TAS showed no significant effect on the viability of WB-F344 cells within the concentration range of 0-12.5 μg/mL, but significantly promoted the proliferation of WB-F344 cells at concentrations ranging from 25-100 μg/mL.

**Supplementary Text 1. The target sequence of AAV8.*Numb*-Exon3.**

ACGGACGAGGACAGGGCCCTGTCTCCTCAGCTTCAGGCACCACCACTGACCTGGGACAGTGAATACCGGTCGCCACCATGAACAAACTACGGCAGAGTTTCAGGAGAAAGAAAGATGTTTACGTCCCAGAGGCCAGCCGTCCACATCAGTGGCAGACAGATGAAGAGGGAGTCCGCACTGGAAAGTGCAGCTTCCCAGTTAAGTACCTTGGCCACGTAGAGGTTGATGAGTCAAGAGGAATGCACATCTGTGAAGATGCCGTCAAAAGATTGAAAGCTGAAAGGAAGTTCTTCAAAGGCTTCTTTGGAAAAACGGGAAAGAAGGCCGTCAAAGCTGTCCTGTGGGTATCCGCCGACGGGCTGAGGGTCGTGGATGAAAAAACTAAGGACCTCATAGTTGACCAGACAATAGAAAAAGTTTCTTTCTGCGCCCCCGATAGGAACTTTGACAGAGCCTTTTCTTACATATGTCGAGATGGCACCACTCGGCGATGGATCTGTCATTGCTTCATGGCTGTCAAAGACACGGGGGAAAGACTGAGCCATGCCGTGGGCTGTGCTTTTGCAGCCTGTTTAGAGCGTAAACAGAAGCGGGAAAAGGAGTGTGGAGTCACTGCTACTTTCGATGCCAGTAGAACCACTTTTACAAGAGAAGGATCATTCCGTGTCACAACTGCCACGGAACAAGCTGAAAGAGAGGAGATCATGAAACAGTTGCAAGATGCCAAGAAAGCTGAGACAGATAAGACCGTTGGTCCATCAGTGGCTCCTGGCAACAGTGCTCCATCGCCGTCCTCTCCCACCTCCCCAACTCTGGATCCCACTGCTTCTTTAGAGATGAACAATCCTCATGCTATCCCACGCCGGCATGCACCAATTGAACAGCTTGCTCGCCAAGGCTCTTTCCGGGGATTTCCTGCTCTTAGCCAGAAGATGTCACCCTTTAAACGCCAGCTGTCCCTACGCATCAATGAGCTGCCTTCCACTATGCAGAGGAAGACTGATTTCCCAATAAAAAACACAGTGCCTGAGGTGGAAGGAGAGGCAGAAAGCATCAGCTCCCTGTGCTCCCAGATCACCAGTGCCTTCAGCACACCCTGTGAGGACCCCTTCTCCTCTGCCCCAATGACCAAACCAGTGACATTAGTGGCACCACAGTCTCCTGTGTTACAAGGGACTGAGTGGGGTCAGTCTTCTGGTGCTGCCTCTCCAGGTCTCTTCCAGGCTGGTCACAGACGCACTCCCTCTGAGGCTGACCGTTGGTTAGAAGAAGTATCAAAAAGTGTGCGGGCCCAGCAGCCGCAGGTCTCAGCCGCCCCTCTGCAGCCAGTTCTGCAGCCTCCTCCGCCCGCTGCCATTGCCCCTCCAGCACCTCCTTTCCAAGGACATGCATTCCTCACTTCTCAGCCTGTGCCAGTGGGTGTGGTCCCACCCCTACAACCAGCCTTTGTCTCTACCCAGTCCTACCCTGTGGCCAATGGGATGCCCTATCCAGCCTCTAATGTGCCTGTAGTGGGCATCACCCCATCCCAGATGGTAGCCAATGTGTTTGGCACTGCAGGCCATCCTCAGGCCACTCATCCACATCAGTCCCCAAGCCTGGCCAAGCAGCAGACATTCCCTCAATATGAGACAAGTAGTGCTACCACCAGTCCCTTCTTTAAGCCTTCTGCTCAGCACCTCAATGGTTCTGCAGCTTTCAATGGTGTAGACAATAGCGGGCTAGTCTCAGGAAACAGACCTGCACAAGTCCCTCCAGGCACCTGCCCAGTGGATCCTTTTGAGGCCCAGTGGGCTGCACTAGAAAGCAAGCCCAAGCAGCGCACCAACCCCTCTCCTACCAACCCTTTCTCCAGTGATGCACAGAAGGCATTTGAAATAGAGCTTGTCTCGAGGGATCCCGCCACCATGGTGAGCAAGGGCGAGGAGCTGTTCACCGGGGTGGTGCCCATCCTGGTCGAGCTGGACGGCGACGTAAACGGCCACAAGTTCAGCGTGTCCGGCGAGGGCGAGGGCGATGCCACCTACGGCAAGCTGACCCTGAAGTTCATCTGCACCACCGGCAAG

**Supplementary Text 2. The target sequence of LV-*Numb-Exon3***

ATGAACAAACTACGGCAGAGTTTCAGGAGAAAGAAAGATGTTTACGTCCCAGAGGCCAGCCGTCCACATCAGTGGCAGACAGATGAAGAGGGAGTCCGCACTGGAAAGTGCAGCTTCCCAGTTAAGTACCTTGGCCACGTAGAGGTTGATGAGTCAAGAGGAATGCACATCTGTGAAGATGCCGTCAAAAGATTGAAAGCTGAAAGGAAGTTCTTCAAAGGCTTCTTTGGAAAAACGGGAAAGAAGGCCGTCAAAGCTGTCCTGTGGGTATCCGCCGACGGGCTGAGGGTCGTGGATGAAAAAACTAAGGACCTCATAGTTGACCAGACAATAGAAAAAGTTTCTTTCTGCGCCCCCGATAGGAACTTTGACAGAGCCTTTTCTTACATATGTCGAGATGGCACCACTCGGCGATGGATCTGTCATTGCTTCATGGCTGTCAAAGACACGGGGGAAAGACTGAGCCATGCCGTGGGCTGTGCTTTTGCAGCCTGTTTAGAGCGTAAACAGAAGCGGGAAAAGGAGTGTGGAGTCACTGCTACTTTCGATGCCAGTAGAACCACTTTTACAAGAGAAGGATCATTCCGTGTCACAACTGCCACGGAACAAGCTGAAAGAGAGGAGATCATGAAACAGTTGCAAGATGCCAAGAAAGCTGAGACAGATAAGACCGTTGGTCCATCAGTGGCTCCTGGCAACAGTGCTCCATCGCCGTCCTCTCCCACCTCCCCAACTCTGGATCCCACTGCTTCTTTAGAGATGAACAATCCTCATGCTATCCCACGCCGGCATGCACCAATTGAACAGCTTGCTCGCCAAGGCTCTTTCCGGGGATTTCCTGCTCTTAGCCAGAAGATGTCACCCTTTAAACGCCAGCTGTCCCTACGCATCAATGAGCTGCCTTCCACTATGCAGAGGAAGACTGATTTCCCAATAAAAAACACAGTGCCTGAGGTGGAAGGAGAGGCAGAAAGCATCAGCTCCCTGTGCTCCCAGATCACCAGTGCCTTCAGCACACCCTGTGAGGACCCCTTCTCCTCTGCCCCAATGACCAAACCAGTGACATTAGTGGCACCACAGTCTCCTGTGTTACAAGGGACTGAGTGGGGTCAGTCTTCTGGTGCTGCCTCTCCAGGTCTCTTCCAGGCTGGTCACAGACGCACTCCCTCTGAGGCTGACCGTTGGTTAGAAGAAGTATCAAAAAGTGTGCGGGCCCAGCAGCCGCAGGTCTCAGCCGCCCCTCTGCAGCCAGTTCTGCAGCCTCCTCCGCCCGCTGCCATTGCCCCTCCAGCACCTCCTTTCCAAGGACATGCATTCCTCACTTCTCAGCCTGTGCCAGTGGGTGTGGTCCCACCCCTACAACCAGCCTTTGTCTCTACCCAGTCCTACCCTGTGGCCAATGGGATGCCCTATCCAGCCTCTAATGTGCCTGTAGTGGGCATCACCCCATCCCAGATGGTAGCCAATGTGTTTGGCACTGCAGGCCATCCTCAGGCCACTCATCCACATCAGTCCCCAAGCCTGGCCAAGCAGCAGACATTCCCTCAATATGAGACAAGTAGTGCTACCACCAGTCCCTTCTTTAAGCCTTCTGCTCAGCACCTCAATGGTTCTGCAGCTTTCAATGGTGTAGACAATAGCGGGCTAGTCTCAGGAAACAGACCTGCACAAGTCCCTCCAGGCACCTGCCCAGTGGATCCTTTTGAGGCCCAGTGGGCTGCACTAGAAAGCAAGCCCAAGCAGCGCACCAACCCCTCTCCTACCAACCCTTTCTCCAGTGATGCACAGAAGGCATTTGAAATAGAGCTTTAG

**Supplementary Text3. The target sequence of LV-*Numb***

ATGAACAAACTACGGCAGAGTTTCAGGAGAAAGAAAGATGTTTACGTCCCAGAGGCCAGCCGTCCACATCAGTGGCAGACAGATGAAGAGGGAGTCCGCACTGGAAAGTGCAGCTTCCCAGTTAAGTACCTTGGCCACGTAGAGGTTGATGAGTCAAGAGGAATGCACATCTGTGAAGATGCCGTCAAAAGATTGAAAGCTGAAAGGAAGTTCTTCAAAGGCTTCTTTGGAAAAACGGGAAAGAAGGCCGTCAAAGCTGTCCTGTGGGTATCCGCCGACGGGCTGAGGGTCGTGGATGAAAAAACTAAGGACCTCATAGTTGACCAGACAATAGAAAAAGTTTCTTTCTGCGCCCCCGATAGGAACTTTGACAGAGCCTTTTCTTACATATGTCGAGATGGCACCACTCGGCGATGGATCTGTCATTGCTTCATGGCTGTCAAAGACACGGGGGAAAGACTGAGCCATGCCGTGGGCTGTGCTTTTGCAGCCTGTTTAGAGCGTAAACAGAAGCGGGAAAAGGAGTGTGGAGTCACTGCTACTTTCGATGCCAGTAGAACCACTTTTACAAGAGAAGGATCATTCCGTGTCACAACTGCCACGGAACAAGCTGAAAGAGAGGAGATCATGAAACAGTTGCAAGATGCCAAGAAAGCTGAGACAGATAAGACCGTTGGTCCATCAGTGGCTCCTGGCAACAGTGCTCCATCGCCGTCCTCTCCCACCTCCCCAACTCTGGATCCCACTGCTTCTTTAGAGATGAACAATCCTCATGCTATCCCACGCCGGCATGCACCAATTGAACAGCTTGCTCGCCAAGGCTCTTTCCGGGGATTTCCTGCTCTTAGCCAGAAGATGTCACCCTTTAAACGCCAGCTGTCCCTACGCATCAATGAGCTGCCTTCCACTATGCAGAGGAAGACTGATTTCCCAATAAAAAACACAGTGCCTGAGGTGGAAGGAGAGGCAGAAAGCATCAGCTCCCTGTGCTCCCAGATCACCAGTGCCTTCAGCACACCCTGTGAGGACCCCTTCTCCTCTGCCCCAATGACCAAACCAGTGACATTAGTGGCACCACAGTCTCCTGTGTTACAAGCTAATGGCACTGACTCAGCCCTCCATGTGCTTACCGCAAAGCCAGCCAGTACTGCTCTAGCACCCGTAGCAATGCCTGTCCGTGAAACCAACCCTTGGGCCCATGCCCCTGATGCTGCTAACAAGGAAATTGCAGCCATACATTCGGGGACTGAGTGGGGTCAGTCTTCTGGTGCTGCCTCTCCAGGTCTCTTCCAGGCTGGTCACAGACGCACTCCCTCTGAGGCTGACCGTTGGTTAGAAGAAGTATCAAAAAGTGTGCGGGCCCAGCAGCCGCAGGTCTCAGCCGCCCCTCTGCAGCCAGTTCTGCAGCCTCCTCCGCCCGCTGCCATTGCCCCTCCAGCACCTCCTTTCCAAGGACATGCATTCCTCACTTCTCAGCCTGTGCCAGTGGGTGTGGTCCCACCCCTACAACCAGCCTTTGTCTCTACCCAGTCCTACCCTGTGGCCAATGGGATGCCCTATCCAGCCTCTAATGTGCCTGTAGTGGGCATCACCCCATCCCAGATGGTAGCCAATGTGTTTGGCACTGCAGGCCATCCTCAGGCCACTCATCCACATCAGTCCCCAAGCCTGGCCAAGCAGCAGACATTCCCTCAATATGAGACAAGTAGTGCTACCACCAGTCCCTTCTTTAAGCCTTCTGCTCAGCACCTCAATGGTTCTGCAGCTTTCAATGGTGTAGACAATAGCGGGCTAGTCTCAGGAAACAGACCTGCACAAGTCCCTCCAGGCACCTGCCCAGTGGATCCTTTTGAGGCCCAGTGGGCTGCACTAGAAAGCAAGCCCAAGCAGCGCACCAACCCCTCTCCTACCAACCCTTTCTCCAGTGATGCACAGAAGGCATTTGAAATAGAGCTTTAG

**Original western blot images**

**
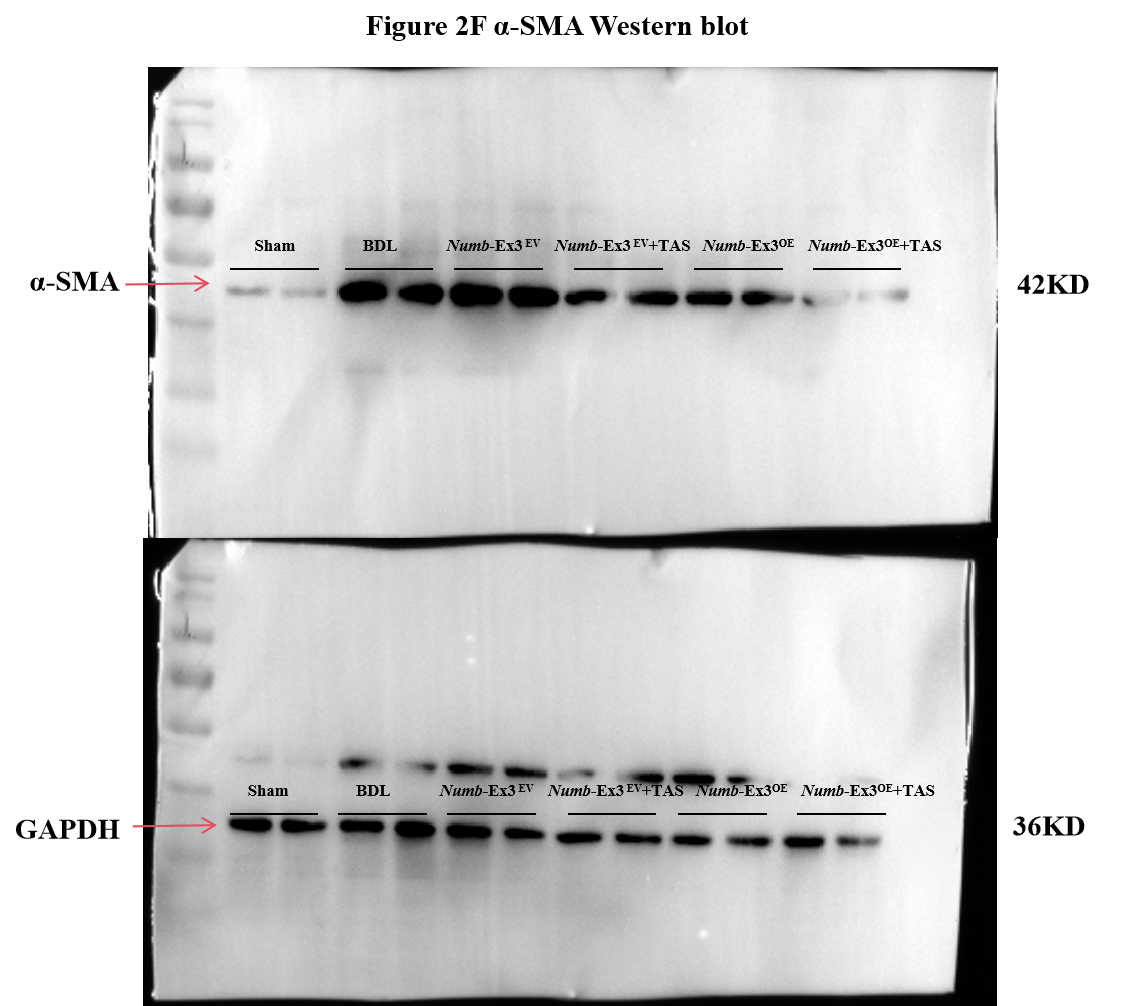
**

**
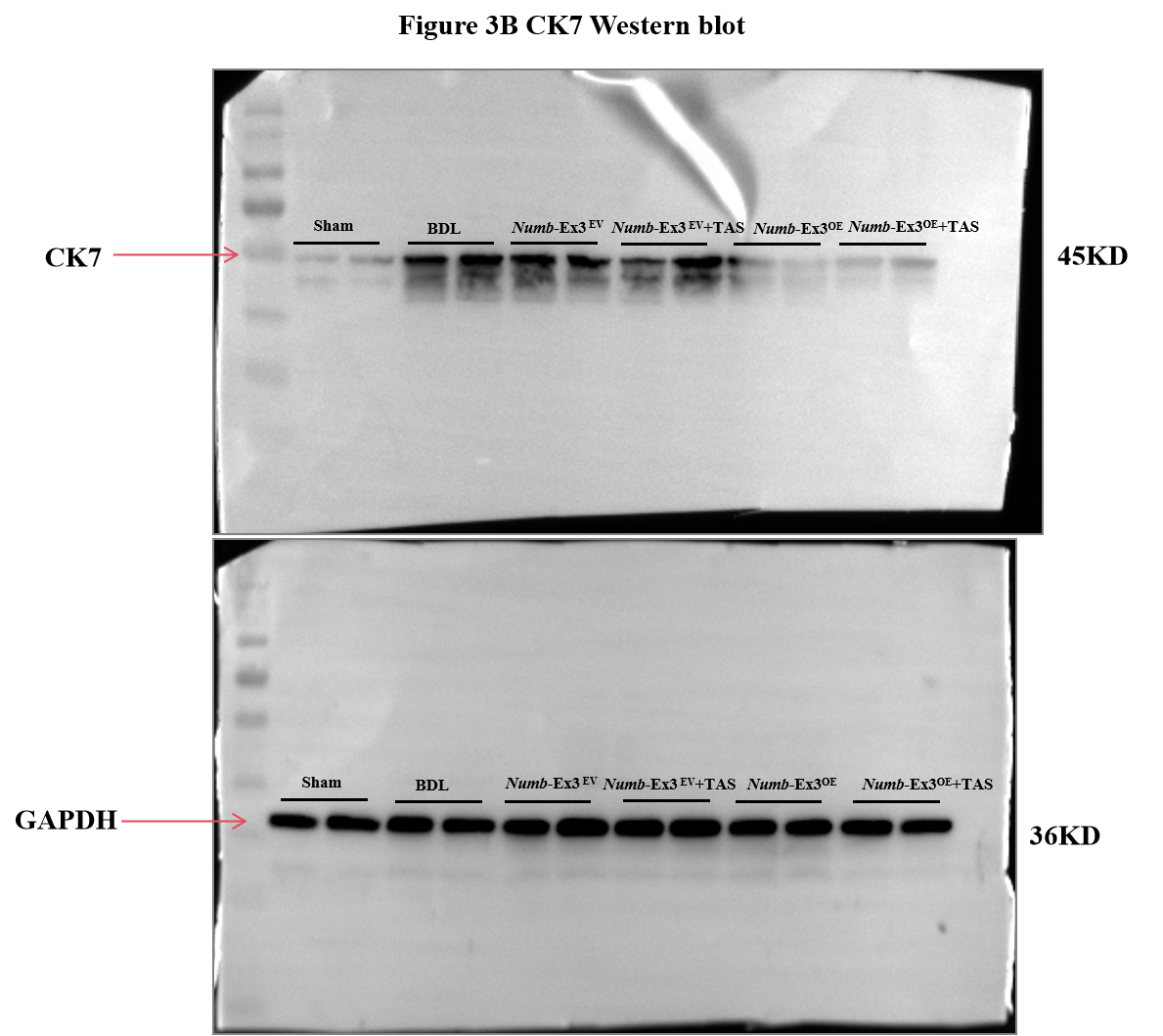
**


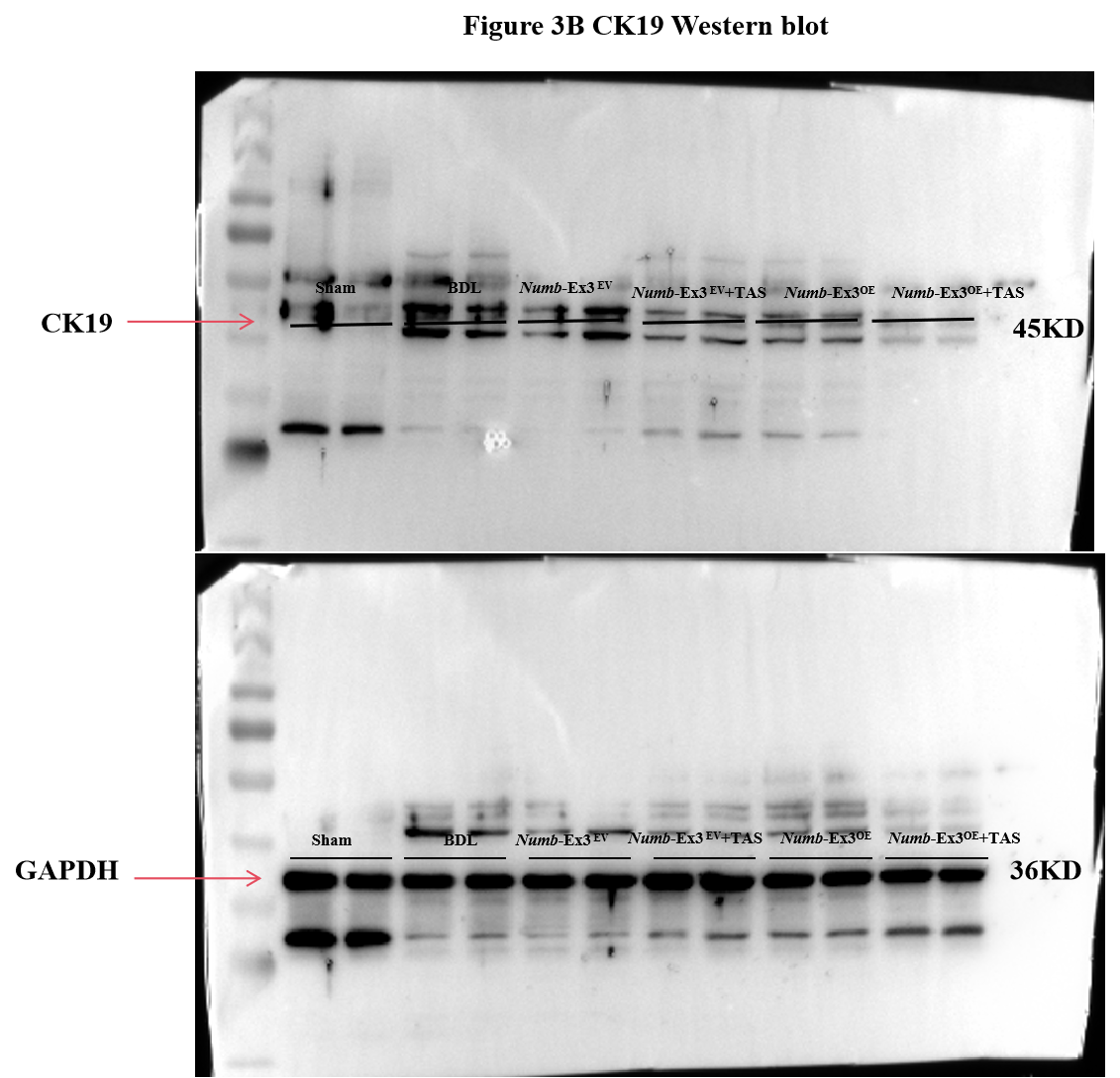


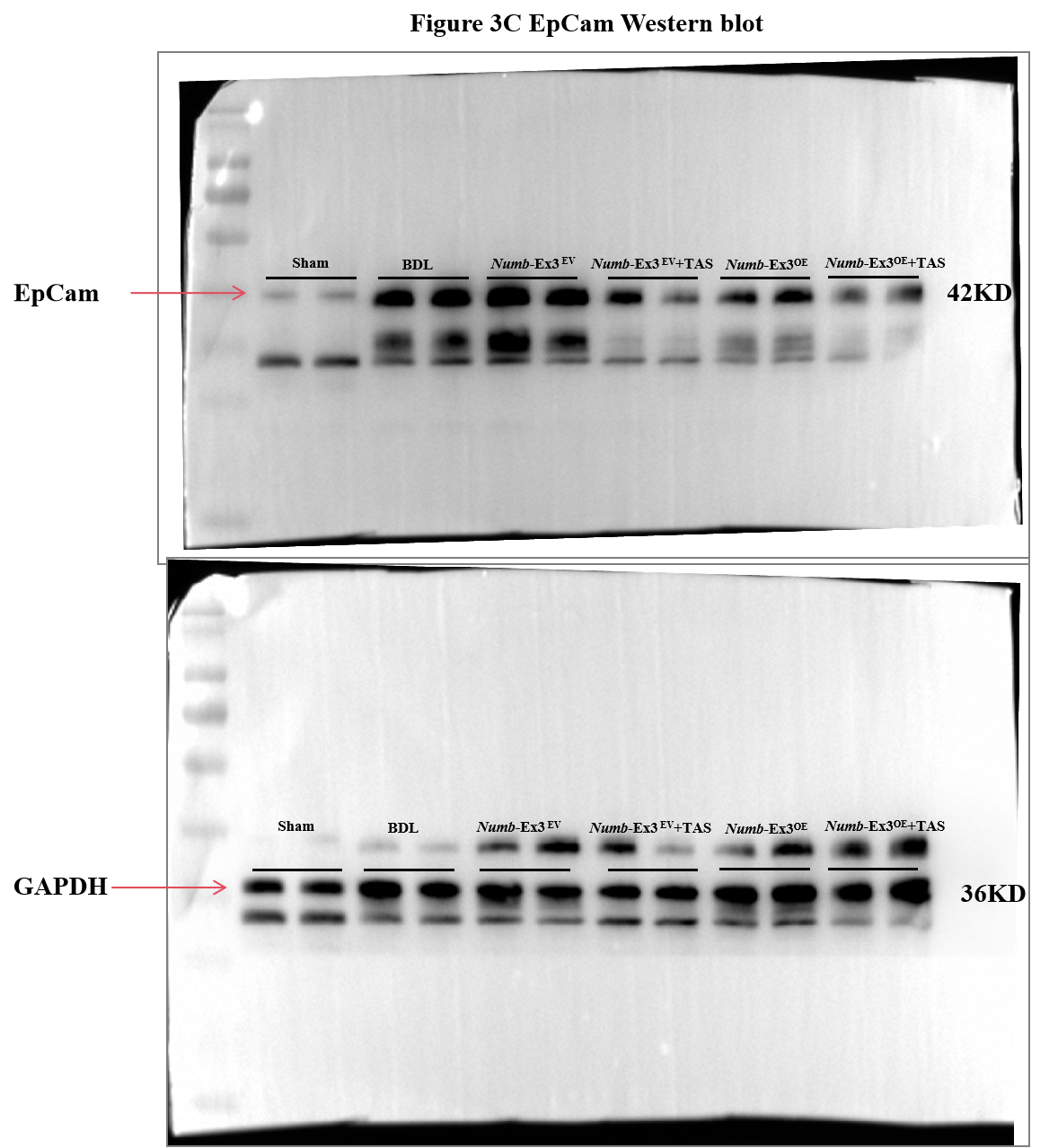


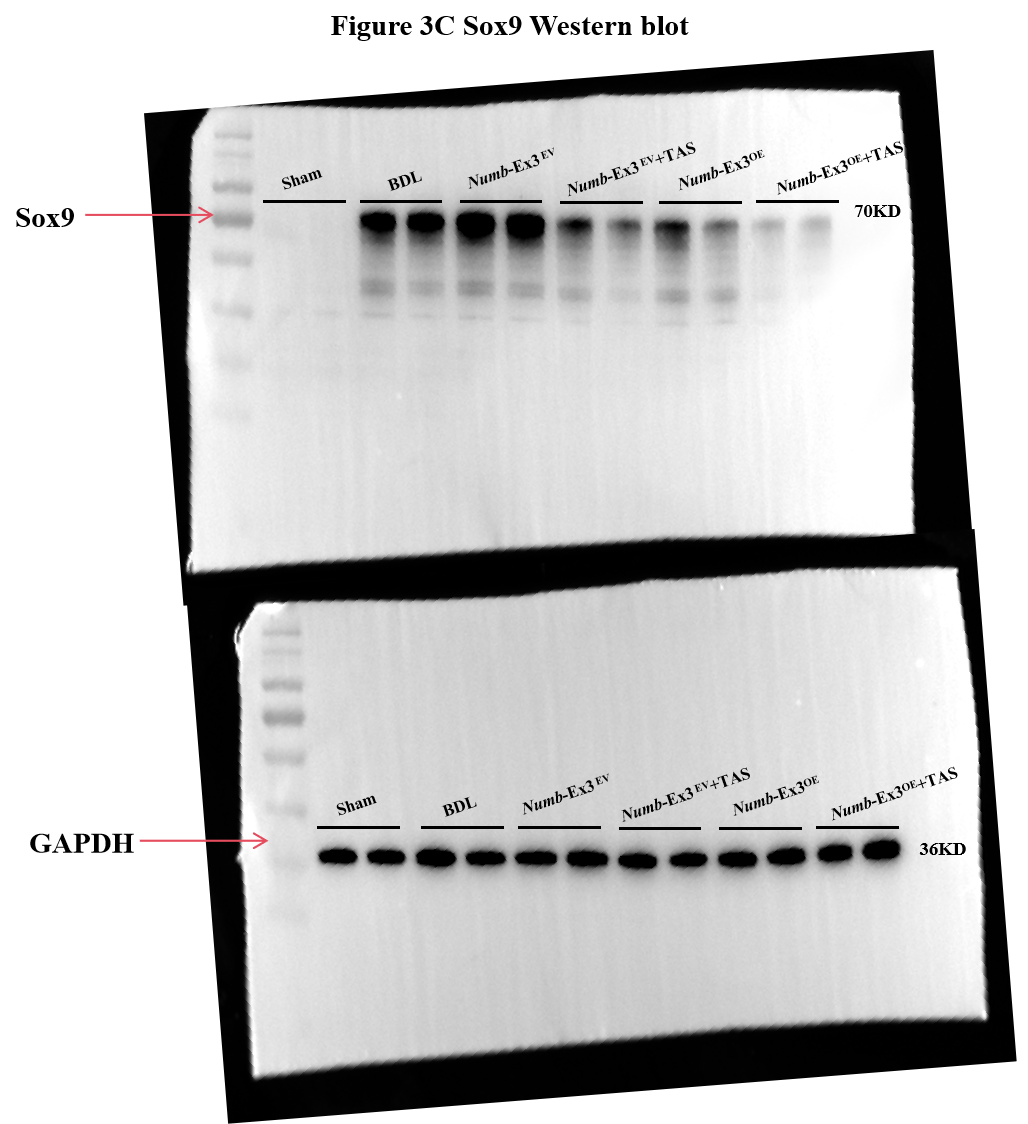


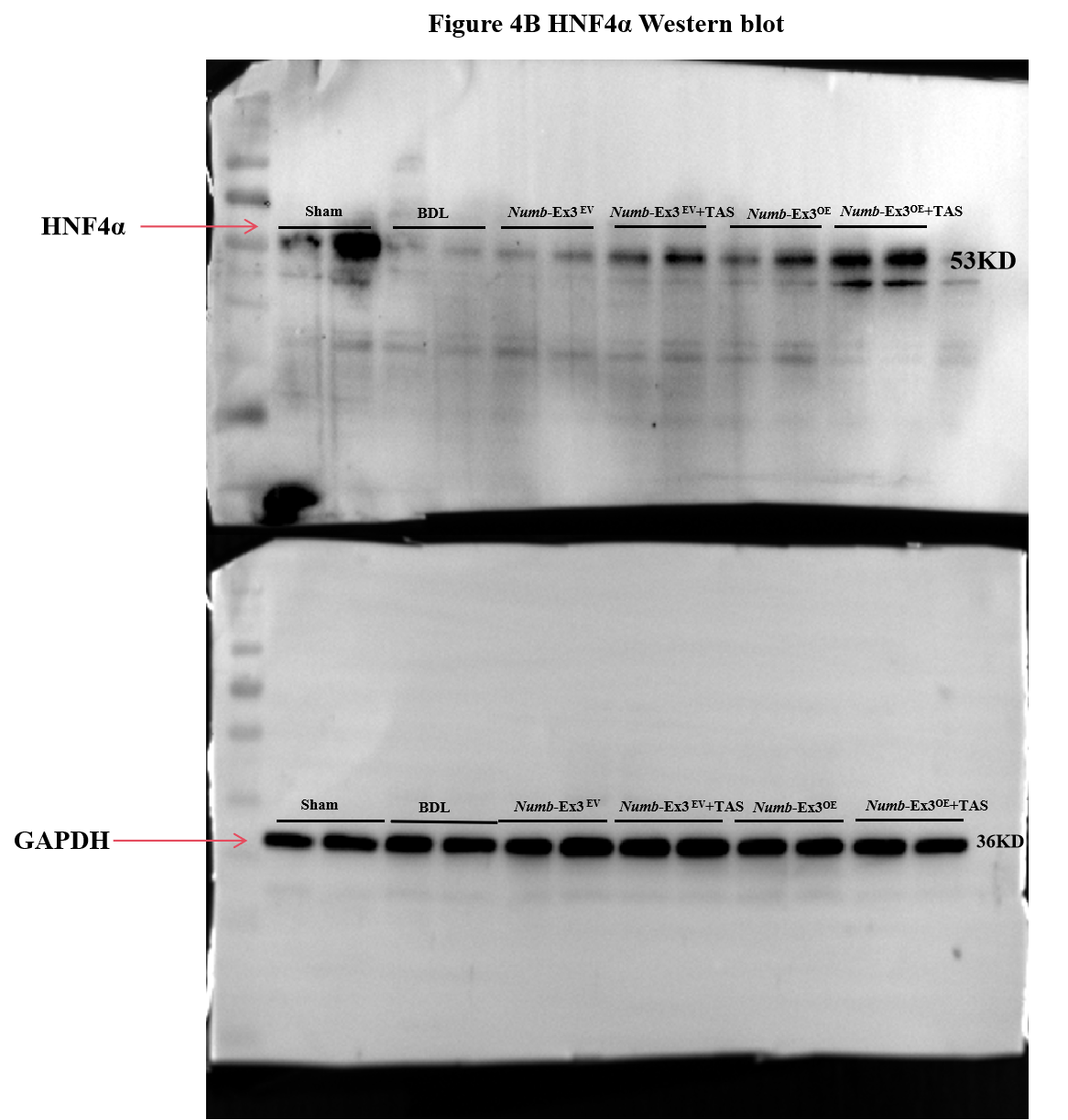


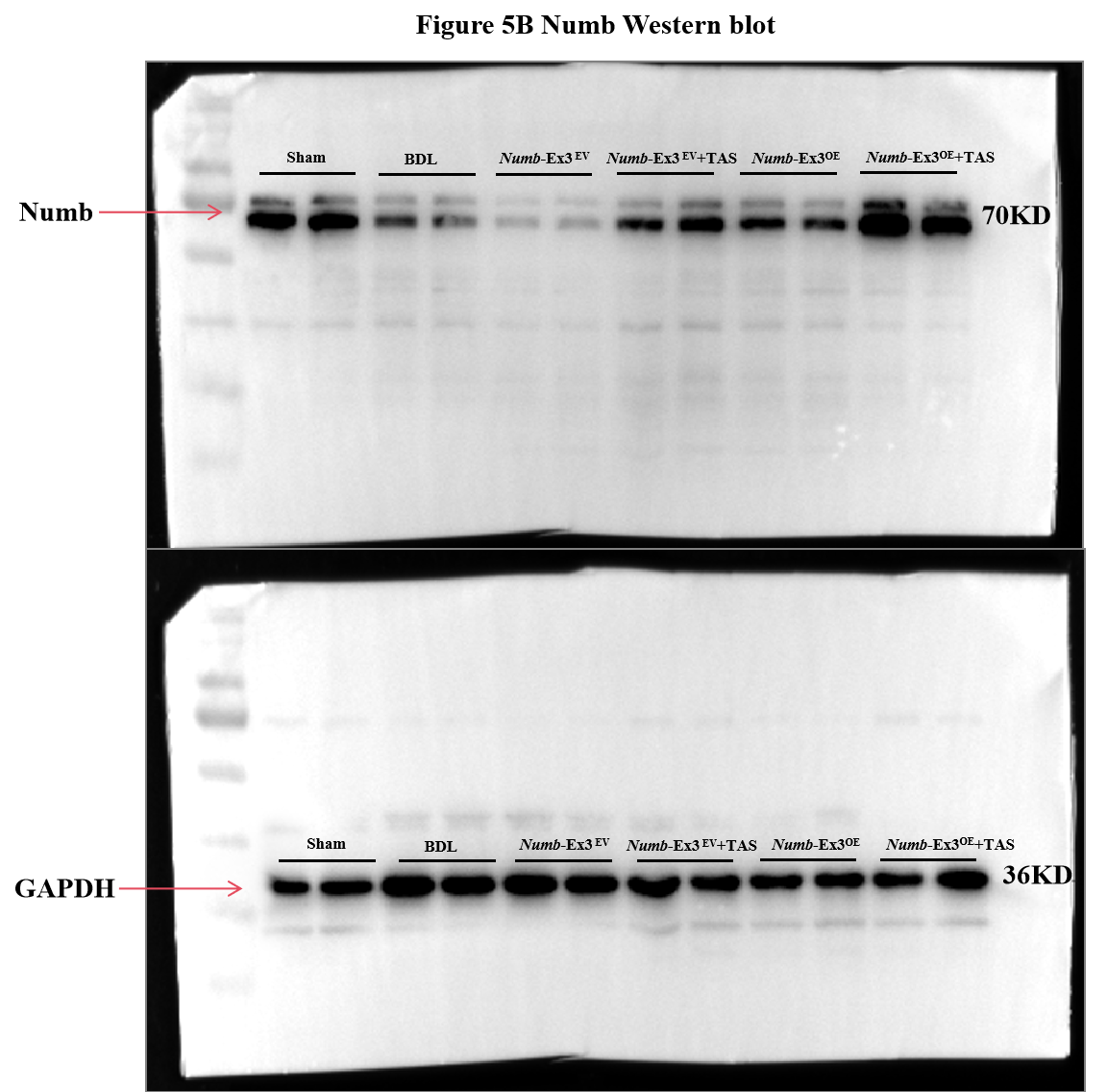


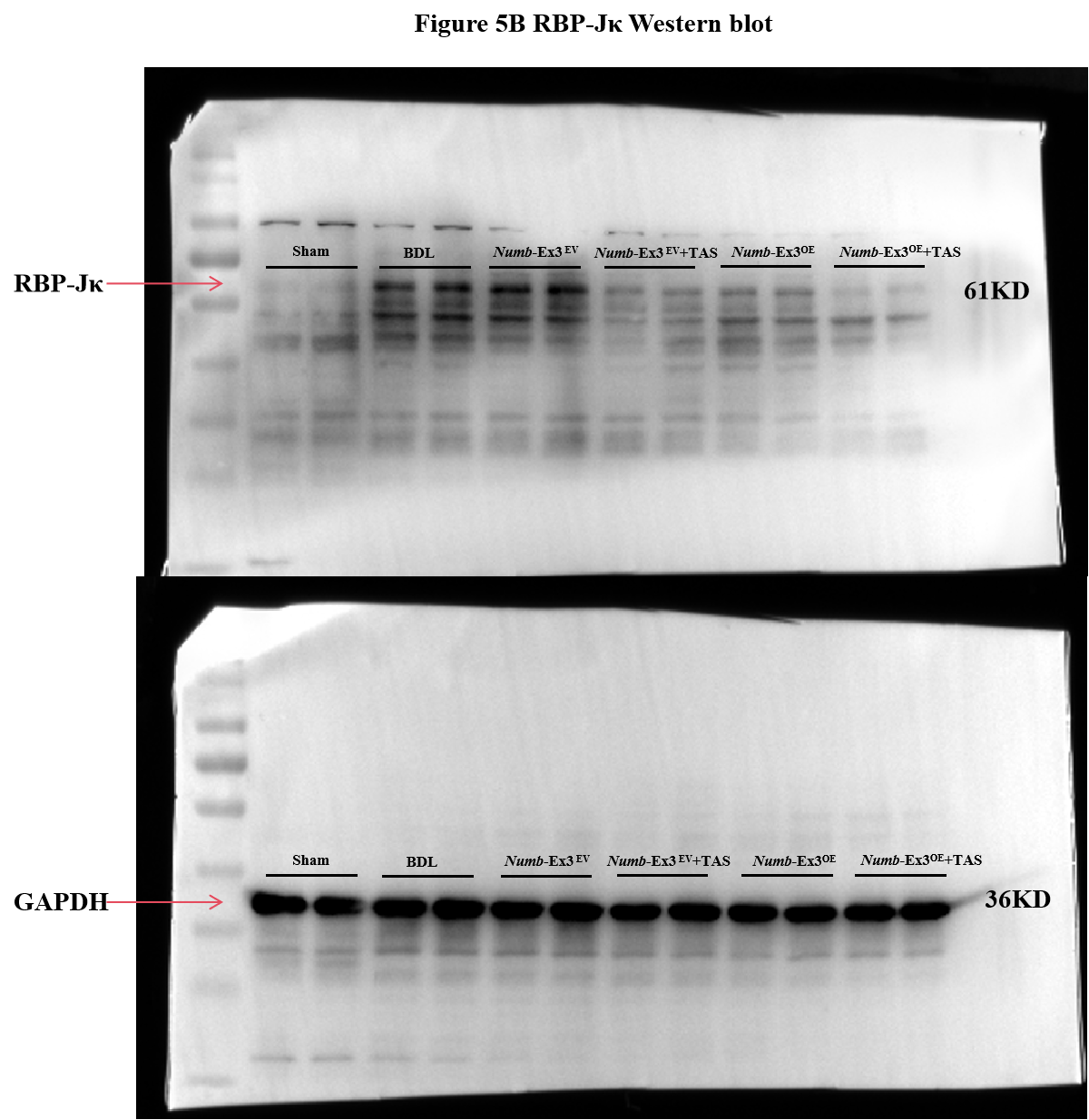


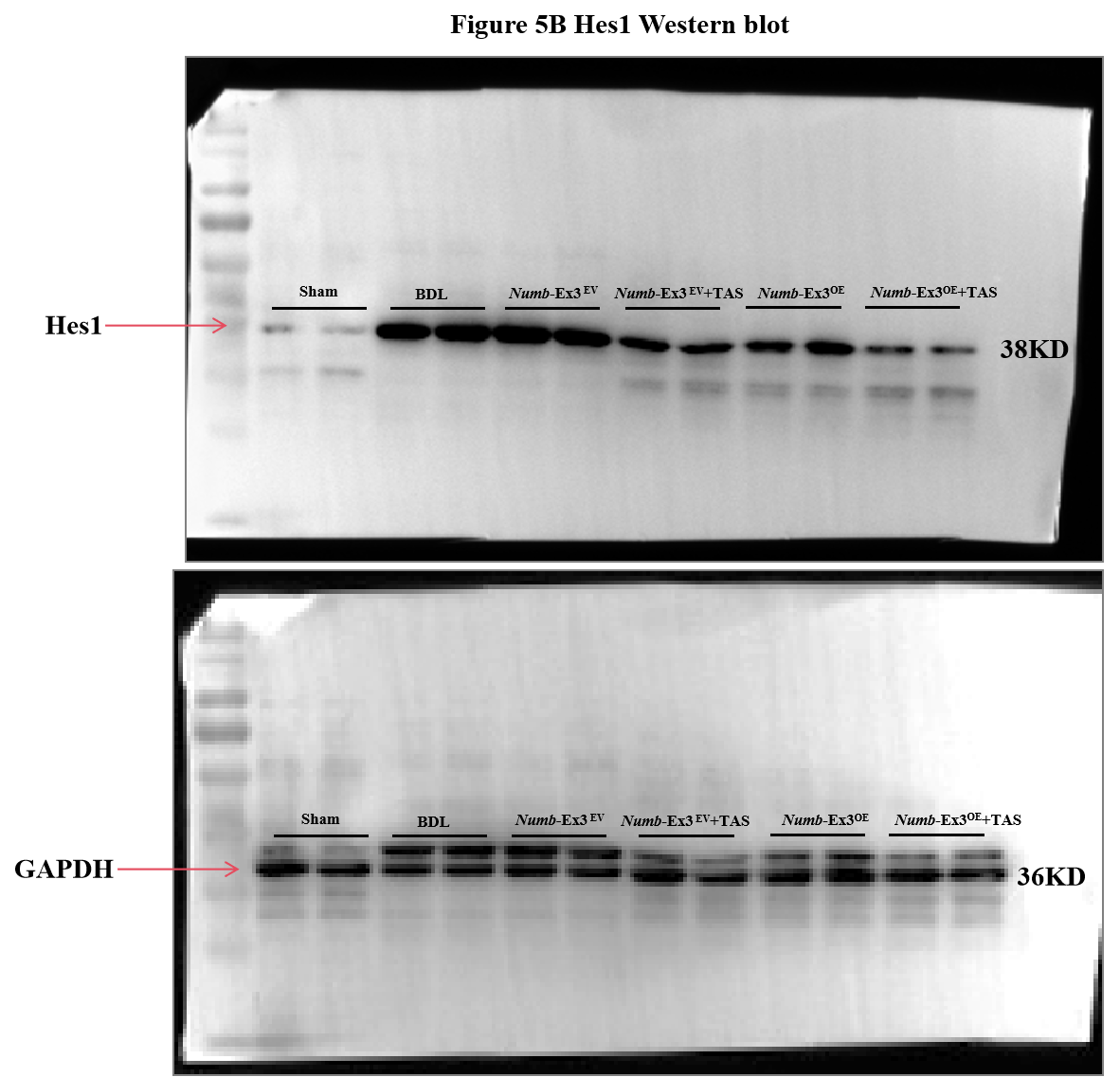


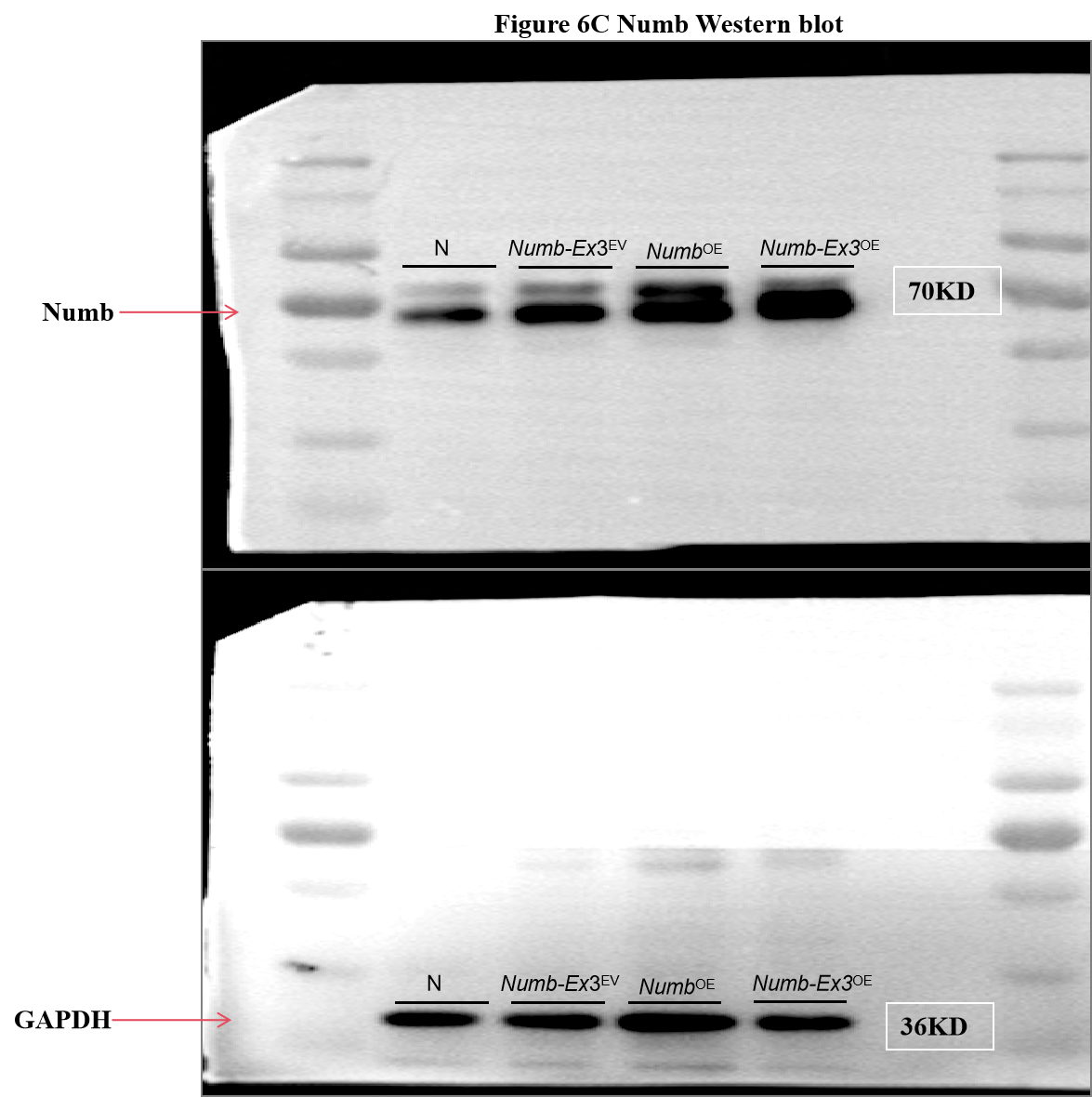


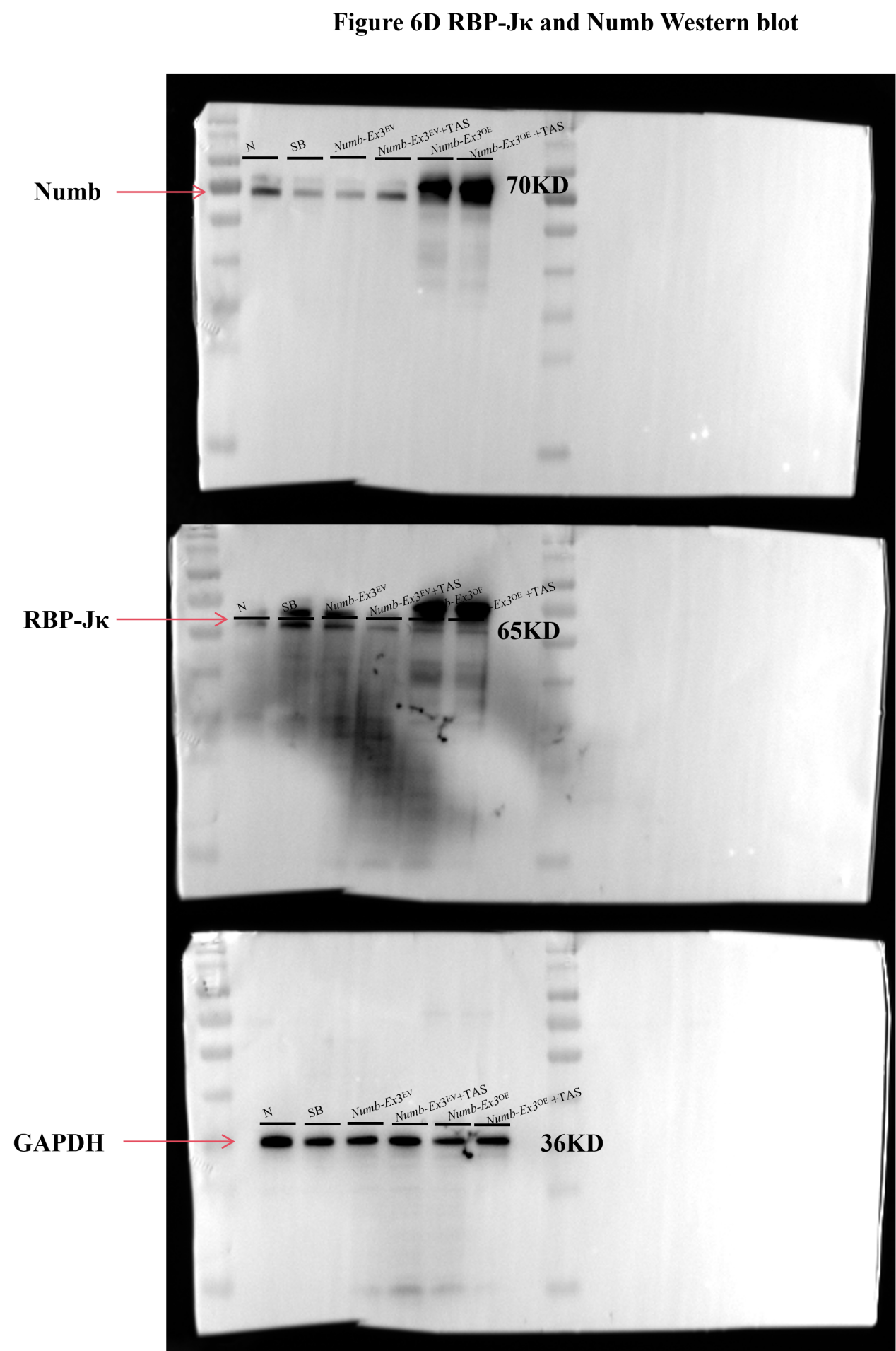


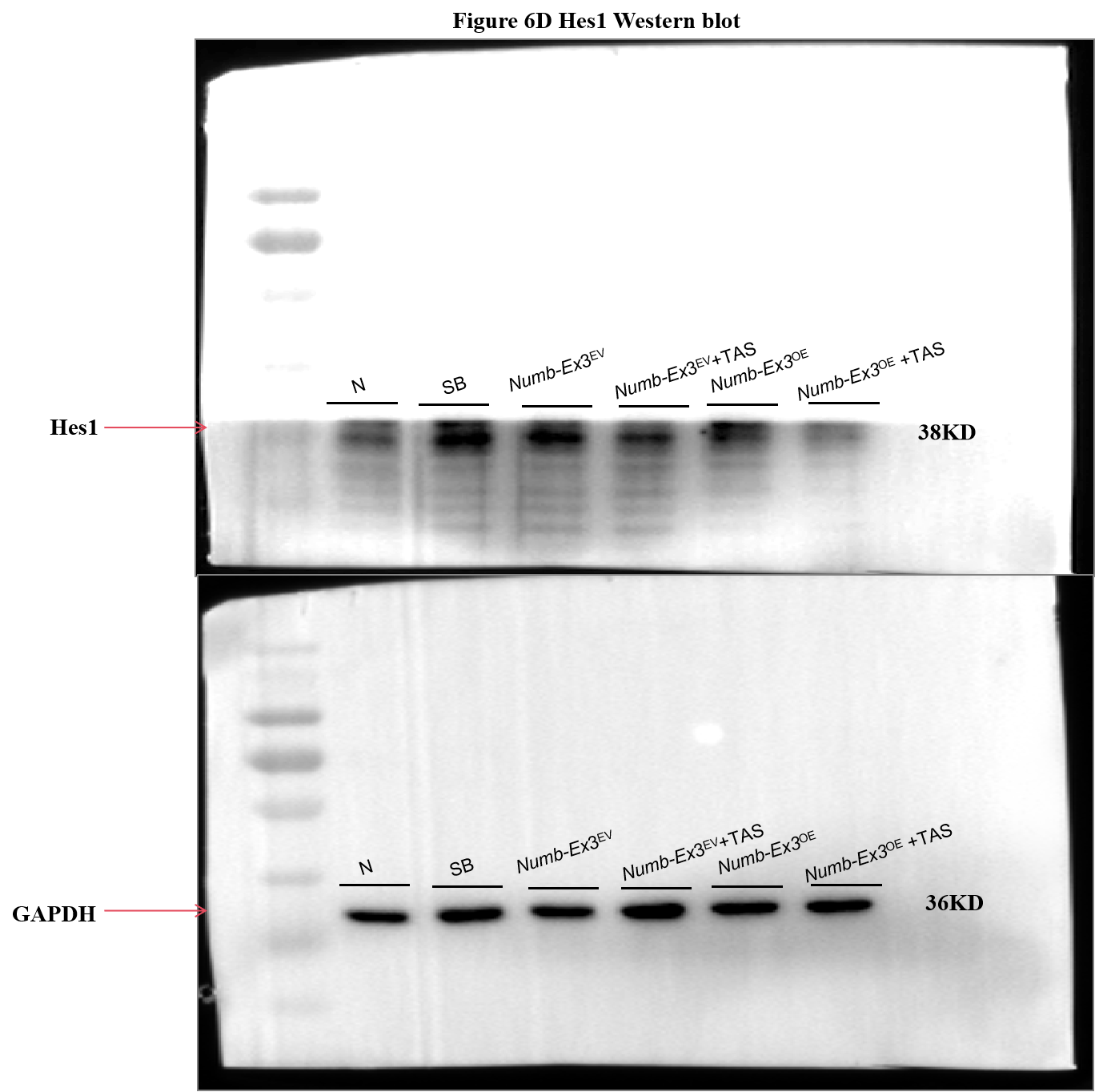


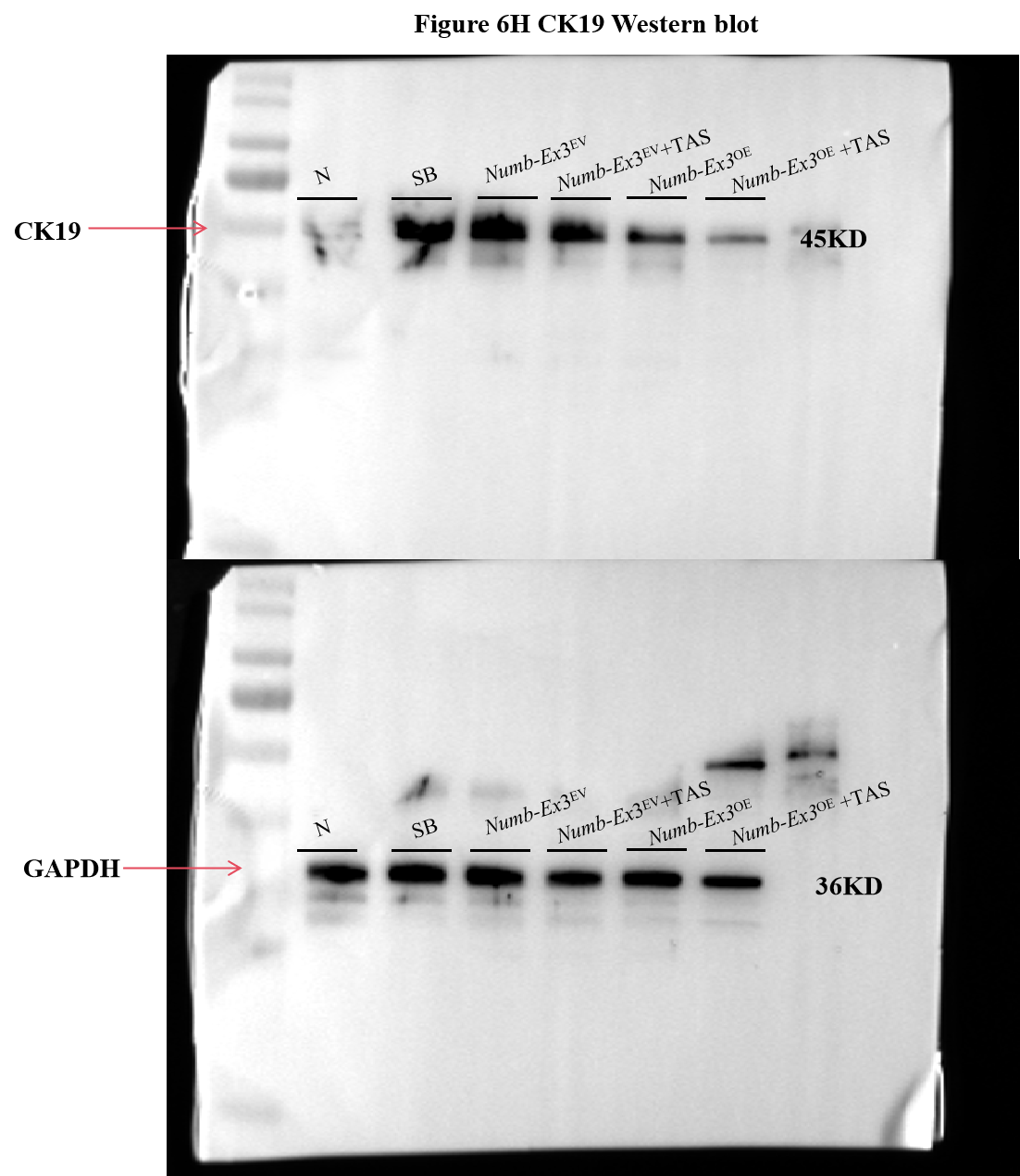

Supplement: Supplementary file 1 [file DataSheet1.docx]
